# ASPIRE: Accurate alternative splicing prediction from limited RNA sequencing data and a minimal gene set

**DOI:** 10.1371/journal.pcbi.1014725

**Published:** 2026-08-28

**Authors:** Ran Eisenberg, Efraim Rahamim, Eli Kopel, Miri Danan-Gotthold, Erez Y. Levanon, Ofir Lindenbaum

**Affiliations:** 1 Alexander Kofkin Faculty of Engineering, Bar-Ilan University, Ramat Gan, Israel; 2 Mina and Everard Goodman Faculty of Life Sciences, Bar-Ilan University, Ramat Gan, Israel; 3 Institute of Nanotechnology and Advanced Materials, Bar-Ilan University, Ramat Gan, Israel; 4 Faculty of Medical & Health Sciences, Tel Aviv University, Tel Aviv, Israel; University of Queensland, AUSTRALIA

## Abstract

Alternative splicing is a fundamental biological mechanism that increases protein diversity and regulates critical cellular processes across eukaryotes. Dysregulation of splicing is implicated in a wide range of diseases, including cancer, neurological disorders, and autoimmune conditions. Accurate prediction of splicing metrics such as percent spliced in (PSI) is therefore essential for understanding splicing regulation and improving disease characterization. However, existing approaches typically require high sequencing depth and are thus poorly suited for low-coverage settings such as single-cell RNA sequencing, where sparse read counts limit reliable splicing analysis. Here, we present ASPIRE (Accurate Splicing Prediction from Limited RNA Sequencing), a deep learning framework for predicting alternative splicing metrics from low-depth RNA-seq gene expression data. ASPIRE infers PSI values from gene expression profiles with limited read coverage and incorporates an embedded feature selection mechanism that identifies a minimal, informative subset of genes relevant to splicing regulation. This design enables accurate prediction while reducing reliance on extensive sequencing and mitigating noise introduced by irrelevant or weakly informative genes. By focusing on biologically meaningful features, including RNA-binding proteins, ASPIRE maintains strong predictive performance even under conditions typical of single-cell transcriptomics. We demonstrate that ASPIRE accurately predicts PSI values across a range of sequencing depths, including those characteristic of single-cell RNA-seq, and performs comparably to or better than existing methods in both simulated and real datasets. By enabling robust expression-based splicing inference from sparse data, ASPIRE facilitates the study of alternative splicing at cellular resolution and provides a practical framework for investigating splicing regulation in development, disease, and heterogeneous cell populations.

## Author summary

Every cell in our body contains the same genetic blueprint, yet they perform vastly different functions. This diversity is partly driven by alternative splicing, a process allowing a single gene to produce multiple protein versions by selectively including or excluding genetic coding segments called exons. This mechanism expands biological complexity and is crucial for regulating cellular behavior, from embryonic development to tissue specialization. To understand these processes, scientists must measure splicing at the single-cell level. However, current technologies often produce “sparse” data, where many biological details are lost because the sequencing depth is too low.

We developed ASPIRE, a deep learning method to overcome these limitations. ASPIRE accurately predicts splicing metrics, such as PSI, from gene expression profiles even when sequencing data is very limited. Using a “feature selection” mechanism, our model identifies a small but critical set of genes that act as indicators for the entire splicing process. Our results demonstrate that ASPIRE maintains strong predictive performance under challenging single-cell conditions, providing reliable expression-based PSI estimates where traditional junction-based methods struggle. By making sense of sparse data, ASPIRE offers a robust framework for exploring gene regulation, helping researchers characterize the molecular diversity that defines complex organisms.

## Introduction

Alternative splicing (AS) is a fundamental process in gene expression regulation, enabling a single gene to produce multiple protein isoforms [1,2]. This process is crucial in cellular function and organismal complexity, from embryonic development and stem cell fate determination to adaptive responses and synaptic plasticity. Dysregulation of AS has been associated with various diseases, including cancer, where it contributes to key aspects of tumor development and progression [3–8].

An accurate analysis of AS is crucial for understanding its role in diseases, cellular function, and organismal complexity. Traditionally, bulk RNA sequencing has been used to study AS. This method provides high sequencing depth while masking heterogeneity among multiple cell types.

Cell-specific splicing events are essential to understanding many physiological states, and bulk RNA sequencing lacks the necessary resolution [9,10]. Single-cell RNA sequencing (scRNA-seq) has revolutionized transcriptome analysis by enabling quantification of gene expression at the single-cell level [11]. This approach has uncovered rare cell types and provided insights into cellular heterogeneity. However, despite these advantages, scRNA-seq typically yields insufficient read depth per cell to capture splicing junctions, thereby limiting its ability to accurately analyze AS.

AS analysis requires sufficient coverage across a gene’s splicing junctions to provide accurate insights. There are scRNA-seq approaches, such as Smart-Seq [12], that capture the entire transcript and enable splicing analysis; however, these methods are costly and, in many cases, do not achieve the coverage required for comprehensive AS analysis. Other droplet-based scRNA-seq methods, like 10X [13], rely on sequencing tags from the 3’/5’ end of a transcript. Although this enables cost-effective quantification of gene expression, it provides limited coverage of the full transcript, including critical splice junctions, hindering accurate AS analysis. Furthermore, achieving the same depth as Bulk RNA-seq, which sequences thousands of cells concurrently, is often prohibitively expensive.

An inherent characteristic of scRNA-seq data is that it yields sparse, high-dimensional gene-expression matrices. The sparsity arises from low sequencing depth per cell, with most genes having zero or very few reads. Sparsity poses significant challenges for AS analysis, as many splicing events may go undetected due to insufficient read coverage. Existing computational tools, such as DeepPSI [14], SCATS [15], and BRIE [16], have demonstrated utility in both bulk RNA-seq and scRNA-seq data. Yet, their performance deteriorates when applied to the sparse coverage characteristic of scRNA-seq data, resulting in missing quantification of alternative splicing events. This limitation hampers efforts to leverage scRNA-seq for high-resolution studies of splicing dynamics, especially in rare cell populations or low-quality samples.

**Fig 1:**
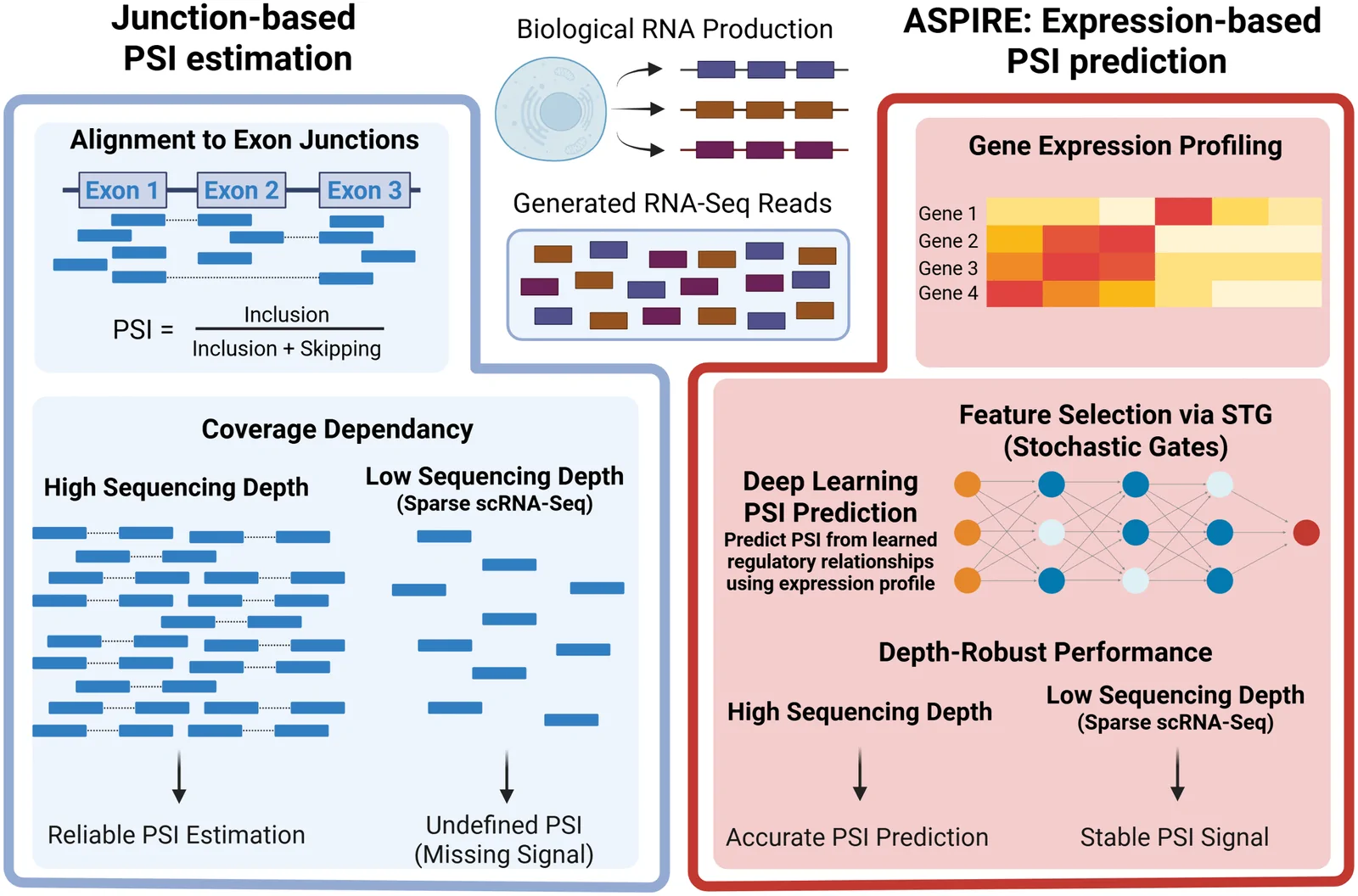
Overview of ASPIRE: expression-based prediction of alternative splicing under sparse sequencing. Conventional junction-based approaches (left) estimate percent spliced-in (PSI) by aligning reads to exon-exon junctions and applying the inclusion/(inclusion + skipping) ratio. This yields reliable PSI at high sequencing depth but breaks down under the sparse coverage typical of single-cell RNA-seq (scRNA-seq), where missing junction reads leave many events with undefined PSI. ASPIRE (right) instead predicts PSI directly from gene expression profiles (TPM): a deep neural network with an embedded Stochastic Gates (STG) feature-selection layer isolates a minimal, informative subset of genes. Because it relies on expression rather than junction coverage, ASPIRE maintains accurate, stable PSI estimates across both high- and low-depth regimes, including scRNA-seq-like sparsity. Created in BioRender. zilbert, **O.** (2026) https://BioRender.com/gyjdaid.

In this study, we aimed to address these limitations by developing ASPIRE (Accurate Splicing Prediction from Limited RNA Sequencing), a deep learning framework that predicts AS metrics from low-depth gene expression data. Fig 1 contrasts conventional junction-based PSI estimation with ASPIRE’s expression-based approach. Our results demonstrate that ASPIRE accurately infers splicing metrics from gene expression where traditional junction-based methods fail, providing a robust complementary approach for studying splicing at single-cell resolution.

## Results

### ASPIRE accurately predicts percent spliced in values at low sequencing depths

We first investigated whether PSI values could be accurately predicted using TPM values derived from bulk RNA sequencing data with ASPIRE. This approach bypasses the need for raw transcript data, which can be challenging to obtain due to privacy concerns. Additionally, it reduces the computational overhead associated with counting reads that align to splice junctions, which is both time-consuming and memory-intensive.

Using the GTEx dataset, we trained ASPIRE to predict PSI from TPM. The ground truth PSI values were calculated from full-depth bulk RNA sequences, as detailed in Equation 1. Full-depth bulk sequencing yields highly reliable PSI estimates that are difficult to obtain at lower sequencing depths, such as those typical of scRNA-seq with 100K reads or fewer.

**Fig 2:**
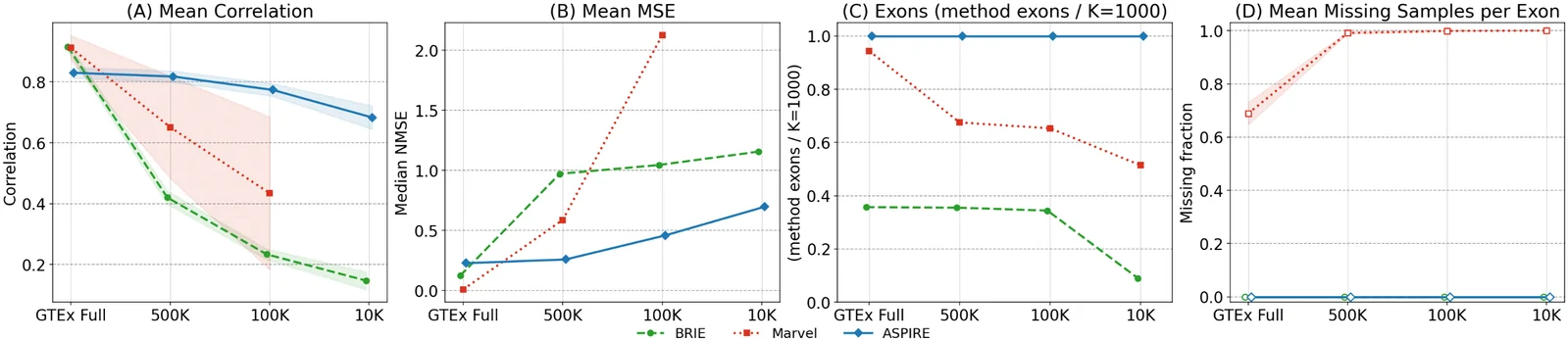
Performance comparison of ASPIRE, BRIE, and MARVEL across sequencing depths (GTEx Full, 500K, 100K, 10K). For panels **(A)**, **(B)**, and **(D)**, metrics are computed on the shared sample–exon intersection across all three methods within each dataset (Full: 708 samples/356 exons; 500K: 708/353; 100K: 708/339; 10K: 708/88). Panel (A) shows mean exon-wise Pearson correlation (95% CI). Panel (B) shows median exon-wise MSE using exons with at least 20 paired observations. Panel (C) reports method-specific valid exons (predicted exons / K = 1000), while panel (D) reports mean missing fraction per exon (95% CI). ASPIRE is the most depth-robust method, achieving the strongest low-depth performance at 500K (corr 0.818, MSE 0.258), 100K (0.774, 0.458), and 10K (0.683, 0.696), whereas BRIE degrades substantially with depth (corr 0.421 to 0.147 from 500K to 10K). Marvel is competitive at Full depth (corr 0.912) but becomes highly sparse at lower depths (usable correlation exons: 19 at 500K, 4 at 100K, 0 at 10K; missing fraction roughly 0.99–1.00), leading to unstable or undefined low-depth estimates.

To evaluate ASPIRE’s performance across sequencing depths, we compared it against BRIE2 [16] and MARVEL [17], two widely used state-of-the-art tools for alternative splicing analysis, on GTEx samples downsampled to progressively lower read depths (Full, 500K, 100K, 10K), focusing on 1,000 exons exhibiting the largest variance in the GTEx dataset. Fig 2 summarizes performance across four complementary axes: mean exon-wise Pearson correlation (Panel A), exon-wise MSE (Panel B), valid exons (Panel C), and missingness (Panel D). Across the low-depth regimes, ASPIRE maintains the strongest predictive accuracy while preserving broad exon coverage, whereas BRIE2 degrades markedly with depth. In contrast, although MARVEL is competitive at full depth, its estimates become highly sparse at reduced depth (near-complete missingness), resulting in unstable or undefined low-depth performance.

**Fig 3:**
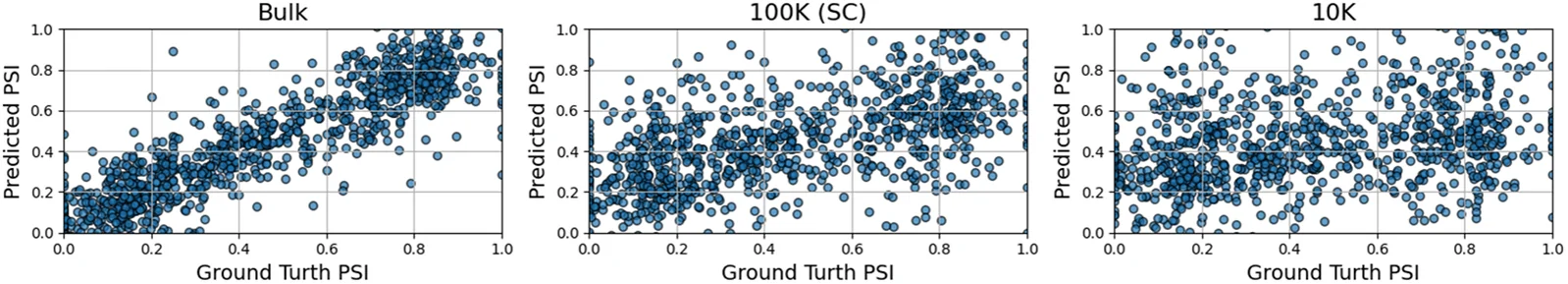
Ground truth and predicted PSI values, for exon *chr1:9756481-9756510* (GRCh38) on gene CLSTN1. The ground-truth PSI was calculated using GTEx full-depth bulk RNA sequencing. The three subplots show our method’s PSI predictions at different depths (Bulk/100K(SC)/10K), where SC denotes single-cell, as 100K is typical for scRNA-seq depth. Correlation and MSE values are computed between $\hat{\text{y}}$ and y: Bulk (correlation = 0.9028, MSE = 0.4100), 100K(SC) (correlation = 0.7820, MSE = 0.6193), and 10K (correlation = 0.5396, MSE = 0.8912).

To further illustrate model performance at the resolution of a specific exon, we focused on exon 3 of the CLSTN1 gene (chr1:9756481–9756510, GRCh38), which has previously been analyzed and shown to have splicing alterations in various cancer types [3]. After training ASPIRE with full-depth bulk sequencing data (typically 30–40M reads), it results in a correlation of 0.9028 and an MSE of 0.4100 between ŷ and y (left subplot of Fig 3); all reported correlation values are statistically significant with p-values smaller than 0.001.

We then evaluated its performance at lower sequencing depths and tested the model using TPM values corresponding to 100K reads (typical of scRNA-seq) and 10K reads.

**Fig 4:**
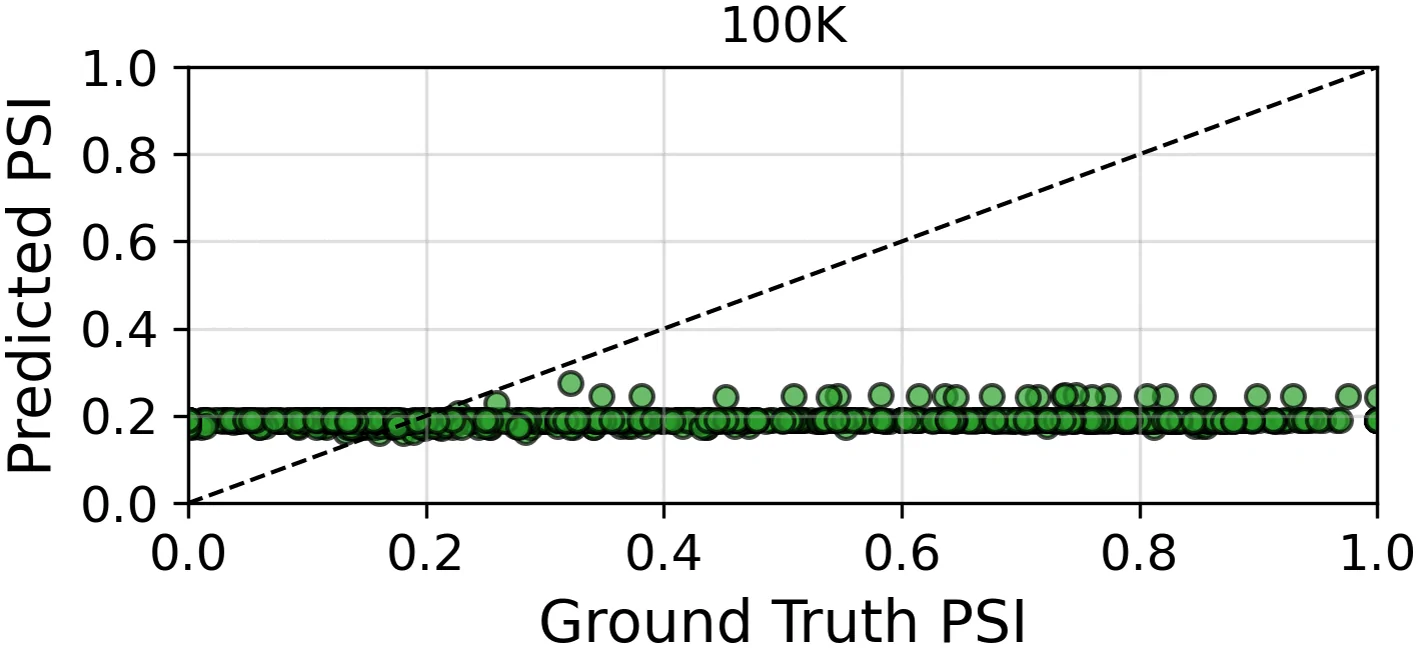
BRIE2-predicted PSI on downsampled GTEx 100K data for the same exemplar exon as in Fig 3. Shown is the agreement between BRIE2-predicted PSI and the bulk-derived GTEx PSI reference (correlation = 0.1946, MSE = 0.1532).

As shown in Fig 3, our model effectively predicted PSI values even when the input TPM was derived from lower sequencing depths. Although the prediction error increased as read counts decreased, the model maintained a reasonable level of accuracy. Specifically, performance metrics indicated a 0.7820 correlation and 0.6193 MSE for 100K reads, and a 0.5396 correlation with 0.8912 MSE for 10K reads (middle and right subplots in Fig 3). For the same exemplar exon, BRIE2 applied to the downsampled GTEx 100K setting shows substantially weaker agreement with the bulk-derived PSI reference (Fig 4; correlation = 0.1946, MSE = 0.1532), highlighting the difficulty of transcripts-based PSI estimation at this depth for this event. This demonstrates the model’s robustness in predicting PSI values across varying sequencing depths.

### Benchmarking ASPIRE against existing PSI estimation tools

To contextualize ASPIRE’s performance relative to existing approaches, we benchmarked it against BRIE2 [16] and MARVEL [17], across three independent datasets (see Methods). These evaluations were designed to assess ASPIRE’s ability to recover accurate PSI values under conditions where traditional junction-based tools often struggle, particularly in low-depth or single-cell–like settings. For each dataset, ASPIRE’s predictions were compared with the corresponding tool-generated PSI values, enabling a direct assessment of predictive accuracy, robustness, and event coverage.

### Benchmarking MARVEL and BRIE2 on downsampled 100K GTEx Data

**Fig 5:**
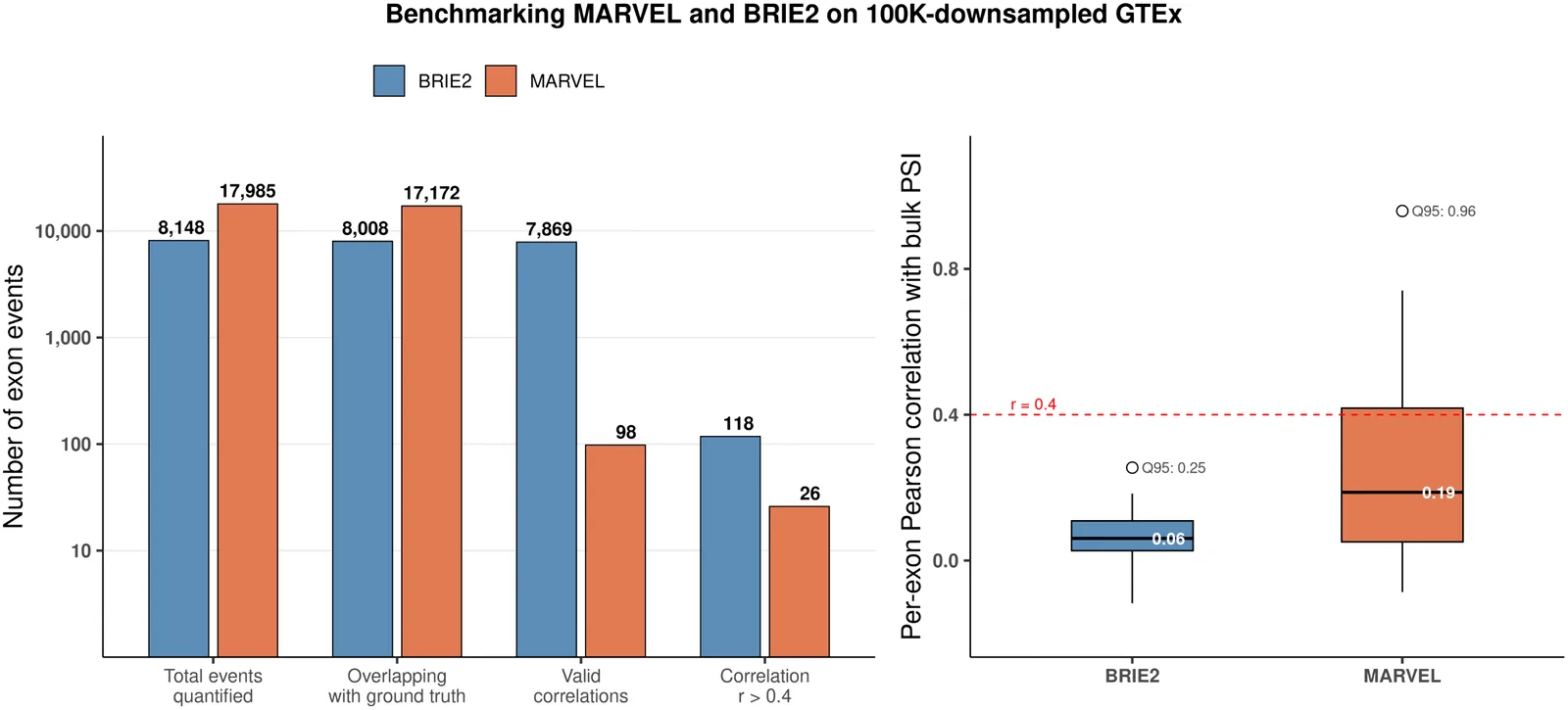
Benchmarking MARVEL and BRIE2 on 100K-downsampled GTEx. Left: Number of exon events retained at each filtering stage for MARVEL and BRIE2, compared against PSI values computed from full-depth bulk RNA-seq (ground truth). Right: Distribution of per-exon Pearson correlations with bulk PSI ground truth. Box spans Q25-Q75; line indicates median; whiskers extend to Q90. Points mark Q95 values. Dashed red line indicates the r = 0.4 threshold used in the text.

We evaluated MARVEL and BRIE2 on the 100K-downsampled GTEx dataset, using PSI values computed from full-depth bulk RNA-seq as the ground truth (Fig 5). MARVEL produced PSI estimates for 17,985 splicing events across 993 samples, of which 17,172 events overlapped with the ground-truth set. However, after applying a minimum threshold of 40 non-NA PSI samples, only 131 events remained suitable for quantitative comparison. Among these, MARVEL generated valid (no NA value) correlations for 98 events, with only 26 exceeding 0.4 (Fig 5, left). MARVEL’s correlation distribution showed highly variable performance, with quantiles ranging from 0.19 (median) to 0.42 (75th percentile), and 0.74/0.96 at the 90th and 95th percentiles, respectively (Fig 5, right). These higher correlations should be interpreted with caution, as many of the corresponding events had relatively few valid samples, which can artificially inflate correlation estimates.

BRIE2 produced PSI estimates for 8,148 splicing events, of which 8,008 overlapped with the ground truth. Unlike MARVEL, BRIE2 yielded valid correlations for nearly all events (7,869 exons), though most showed weak agreement with bulk PSI. Only 118 events exceeded a correlation of 0.4 (Fig 5, left). BRIE2’s correlation distribution was generally low, with a median of 0.06, a 75th percentile of 0.11, and a 95th percentile of 0.25; only the top 1% of events reached correlations above 0.45 (Fig 5, right).

### Benchmarking on single-cell RNA-seq data from multiple sclerosis (Mouse; GSE113973)

We next evaluated ASPIRE on a real single-cell RNA-seq dataset from a mouse model of multiple sclerosis (GSE113973), for which BRIE2 previously reported PSI values. Because BRIE2 provided published PSI estimates for this dataset, we used these values as a reference to assess the level of agreement between ASPIRE and BRIE2. BRIE2 quantified PSI for 3,780 splicing events, whereas MARVEL reported 8,293 events, of which 2,417 contained at least one real PSI value.

ASPIRE was trained on all exons using TPM values derived from the matched single-cell expression profiles. When evaluated against the BRIE2-derived reference values, ASPIRE achieved meaningful correlations for only a subset of events; among these, the top 10 highest correlations ranged from 0.4725 to 0.7901. Examination of the highest-performing events revealed clear linear relationships between ASPIRE-predicted PSI and BRIE2-reported PSI, with several events showing strong concordance even in the presence of substantial variability in single-cell expression levels (S1 Fig). These examples demonstrate that ASPIRE predictions can achieve strong concordance with BRIE2-derived PSI estimates in real single-cell data when sufficient biological signal is present. At the same time, the relatively small number of events exhibiting strong agreement highlights the intrinsic challenges of predicting PSI under the extreme sparsity and technical noise characteristic of single-cell RNA-seq.

### Benchmarking on human HiPSC differentiation data (PRJEB15062)

Lastly, we evaluated ASPIRE on a human single-cell RNA-seq dataset profiling various stages of hiPSC differentiation toward definitive endoderm (PRJEB15062), for which MARVEL previously reported PSI values. Because MARVEL provided published PSI estimates for this dataset, we used these values as a reference to assess the level of agreement between ASPIRE and MARVEL. MARVEL quantified PSI for 20,509 splicing events, of which 17,783 contained at least one real PSI value across samples.

ASPIRE was trained on the 262 splicing events shared between the MARVEL and BRIE2 outputs, and for each event, we computed the correlation between ASPIRE-predicted PSI values and the MARVEL-derived reference values. Using the same evaluation protocol, we computed the corresponding correlations for BRIE2. An exon was considered a “win” for a given method if its correlation with the MARVEL reference exceeded *r* > 0.4 and was higher than that of the competing method.

Under this criterion, BRIE2 achieved higher correlation for 126 events, whereas ASPIRE achieved higher correlation for 136 events. Representative examples illustrating cases in which either ASPIRE or BRIE2 more closely matched the MARVEL-derived PSI values are shown in S2 Fig. These examples highlight that each method captures complementary subsets of splicing events, reflecting differences in the information they leverage.

Importantly, ASPIRE achieves performance comparable to BRIE2, despite relying solely on gene expression features rather than splice-junction reads. This indicates that expression-based prediction can capture meaningful splicing-related signal even in the absence of junction-level evidence. The comparable level of agreement across events suggests that ASPIRE provides a complementary expression-based approach for PSI estimation in settings where junction-based approaches are limited by sparse coverage or technical constraints.

### Consistent evaluation across a defined gene panel

**Fig 6:**
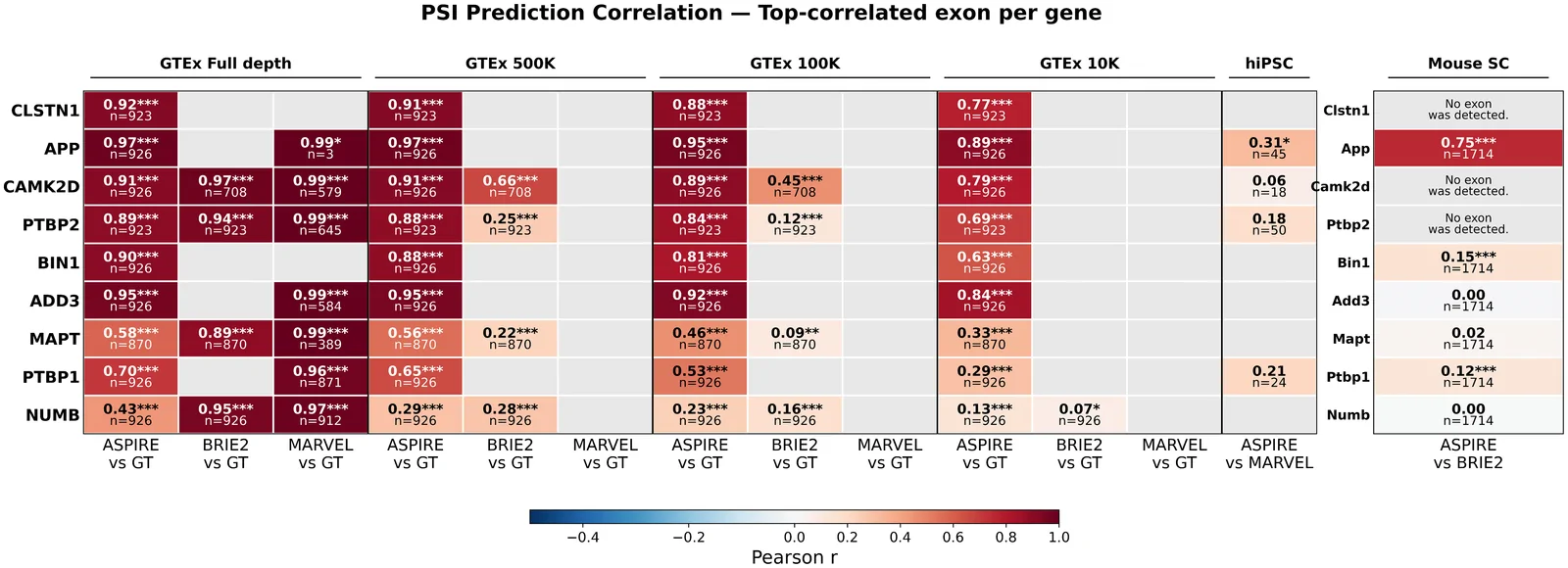
PSI prediction correlation heatmap for the chosen gene panel (best exon per gene). Heatmap showing the Pearson correlation of the best-performing cassette exon per gene across datasets and methods. Rows correspond to nine genes, labelled by gene symbol; the exon shown for each gene is listed at the end of this legend. Columns are grouped by dataset and sequencing depth: GTEx (Full, 500K, 100K, 10K) with three sub-columns per depth (ASPIRE vs ground truth, BRIE2 vs ground truth, MARVEL vs ground truth), plus two single-cell columns (ASPIRE vs MARVEL for the hiPSC dataset, ASPIRE vs BRIE2 for the mouse MS dataset). Cell text shows the Pearson r value with significance annotation (* p < 0.05, ** p < 0.01, *** p < 0.001) and sample count **(n)**. Cell color reflects the Pearson r value. Grey cells in the BRIE2 and MARVEL columns denote exon–dataset combinations for which the respective tool did not report a PSI value, so no correlation could be computed; in the Mouse SC panel, grey cells labelled ‘No exon was detected’ mark genes for which no cassette exon was detected in BRIE2 output. For each gene, the cassette exon shown is, in most cases, the one with the highest absolute correlation at full GTEx depth. The exon shown for each gene is: CLSTN1, chr1:9756480-9756511; APP, chr21:25997359-25997417; CAMK2D, chr4:113455725-113455822; PTBP2, chr1:96806418-96806453; BIN1, chr2:127057472-127057602; ADD3, chr10:110132304-110132401; MAPT, chr17:45971858-45971946; PTBP1, chr19:805491-805570; NUMB, chr14:73284080-73284375. Mouse (Mouse SC) exons: App, chr16:85043576-85043743; Bin1, chr18:32431671-32431742; Add3, chr19:53232356-53232433; Mapt, chr11:104321063-104321159; Ptbp1, chr10:79860117-79860194; Numb, chr12:83855440-83855524; no cassette exon was detected for Clstn1, Camk2d or Ptbp2 in BRIE output. Coordinates refer to human GRCh38/hg38, mouse GRCm38/mm10. Exon coordinates and full per-exon results are provided in S1 Table and S2 Table, respectively.

To evaluate ASPIRE on a pre-defined, biologically motivated gene set, we selected nine genes with well-characterized cassette exon events: CLSTN1, PTBP1, PTBP2, ADD3, MAPT, APP, CAMK2D, BIN1, and NUMB. This panel includes RNA-binding proteins that regulate splicing (PTBP1, PTBP2), classic targets of tissue-specific alternative splicing (MAPT, APP, ADD3, CAMK2D, BIN1, NUMB), and the exemplar gene from earlier analyses (CLSTN1). For each gene, we identified cassette exon events in human (hg38) and mouse (mm10) using VastDB, and evaluated ASPIRE alongside BRIE2 and MARVEL across four GTEx sequencing depths (Full, 500K, 100K, 10K) and two single-cell datasets (human hiPSC differentiation, mouse multiple sclerosis). The full list of exon coordinates is provided in S1 Table, and per-exon correlation results in S2 Table. Fig 6 summarizes the results at the gene level, showing the Pearson correlation of the best-performing cassette exon per gene; grey cells in the BRIE2 and MARVEL columns indicate exon–dataset combinations for which the respective tool did not report a PSI value, so no correlation could be computed. At full GTEx depth, ASPIRE achieved strong correlations for seven of nine genes, including APP (r = 0.98), ADD3 (r = 0.96), CAMK2D (r = 0.91), CLSTN1 (r = 0.92), BIN1 (r = 0.90), PTBP2 (r = 0.90), and PTBP1 (r = 0.71). ASPIRE’s performance decreased only gradually with sequencing depth, consistent with the depth-dependent trends reported above, remaining significant for these genes across all four depths. MAPT (r = 0.58) and NUMB (r = 0.44) showed more moderate correlations. At full depth, BRIE2 and MARVEL also produced strong correlations for several genes, in a number of cases matching or exceeding ASPIRE. For example, for MAPT and NUMB, BRIE2 reached r = 0.89 and r = 0.95 (compared with ASPIRE’s r = 0.58 and r = 0.44), with MARVEL comparably high. As sequencing depth decreased, however, their agreement degraded far more steeply than ASPIRE’s: BRIE2 correlations fell sharply (e.g., PTBP2 from r = 0.94 at full depth to r = 0.26 at 500K and r = 0.12 at 100K) and were largely absent by 10K, while MARVEL returned no PSI estimates below full depth. This behaviour is expected for junction-based quantification, which depends on junction-spanning reads and yields fewer usable estimates as coverage declines; by predicting PSI from expression instead, ASPIRE remained accurate in this sparse, low-depth regime, which is the setting it is designed for. In the single-cell datasets, the strongest agreement was observed for APP (r = 0.32 vs MARVEL in hiPSC; r = 0.75 vs BRIE2 in mouse), while most other genes showed weaker concordance. S3 Fig presents the full per-exon results. Within each gene, individual exons exhibited substantial variability in prediction accuracy. For example, ADD3 contained one exon with r = 0.96 and another with r = 0.13, and PTBP1 ranged from r = 0.71 to near zero across its four exons, indicating that not all cassette exon events are equally predictable from expression profiles.

### Non-linear methods achieve the best performance for PSI prediction

Accurately predicting PSI values from gene expression data is challenging because of the complex relationships between gene expression patterns and splicing outcomes. To address this challenge, we evaluated multiple methods, to determine whether nonlinearity plays a significant role.

Several existing methods designed for PSI prediction from RNA-seq data, including BRIE [16], Outrigger [18], and MARVEL [17], were unable to generate predictions for exon 3 of the CLSTN1 gene (chr1:9756481–9756510, GRCh38) in the 100K GTEx dataset. This inability suggests limitations in their applicability in certain cases, underscoring the need for a more robust, generalizable approach. To address this, we compared four methodological approaches: LASSO, standard Multi-Layer Perceptron (MLP), and our proposed method, ASPIRE.

**Fig 7:**
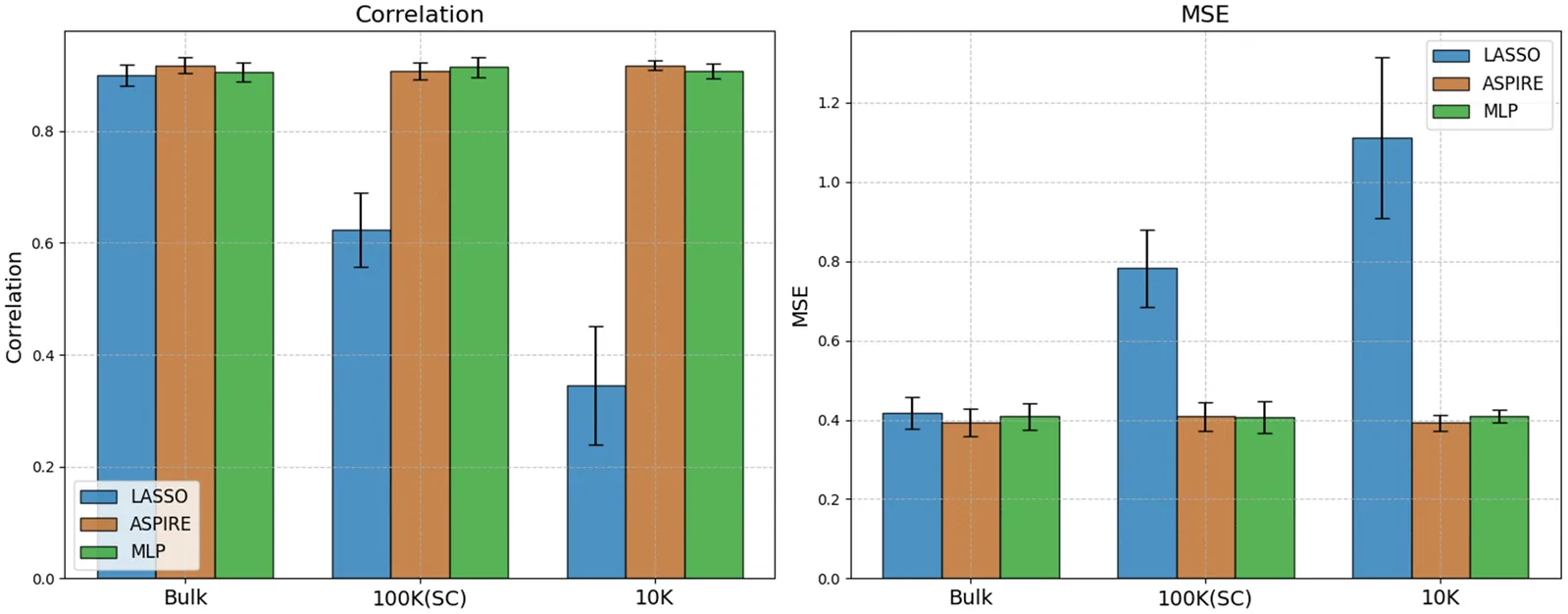
Correlation/MSE between predicted and real PSI values for different methods: LASSO, standard Multi-Layer Perceptron (MLP), and our proposed method, ASPIRE. Correlation and MSE values are computed between $\hat{\text{y}}$ and y. Across TPM based on different depths (Bulk/100K(SC)/10K), where SC stands for single-cell, as 100K is associated with typical scRNA-seq depth. Both non-linear methods, MLP and ASPIRE, outperform the linear method, LASSO. Specifically for Bulk sequencing depth, ASPIRE achieved the highest correlation of 0.918 ± 0.015 and the lowest MSE of 0.034 ± 0.034, while MLP showed similar performance with a correlation of 0.906 ± 0.017 and MSE of 0.034 ± 0.034. For the linear method, LASSO achieved a correlation of 0.900 ± 0.019 and an MSE of 0.040 ± 0.040.

The comparative analysis revealed that non-linear methods (MLP and ASPIRE) consistently outperformed the linear method (LASSO), in predicting PSI values, as shown in Fig 7. Specifically for Bulk sequencing depth, ASPIRE achieved the highest correlation of 0.918 ± 0.015 and the lowest MSE of 0.034 ± 0.034, while MLP showed similar performance with a correlation of 0.906 ± 0.017 and an MSE of 0.034 ± 0.034. For the linear method, LASSO achieved a correlation of 0.900 ± 0.019 and an MSE of 0.040 ± 0.040.

The superior performance of non-linear methods suggests that the relationship between gene expression and PSI values is inherently complex and cannot be adequately captured by linear models alone. This finding aligns with our biological understanding of splicing regulation, in which multiple factors and their interactions collectively influence splice-site selection and exon-inclusion rates [19–21]. Furthermore, the comparable performance of MLP and ASPIRE indicates that our feature selection approach preserves predictive power while potentially improving interpretability by identifying key regulatory genes.

### ASPIRE enables accurate prediction of exon PSI using only a small gene set

The use of TPM values has proven effective in predicting PSI values, which are critical for understanding splicing regulation and its biological implications. However, using all available gene expression data may introduce noise and a risk of overfitting, as many genes have little to no biological relevance to the splicing of specific exons. In addition, identifying the key regulatory genes that drive alternative splicing of particular exons remains a crucial biological challenge. To address this, we evaluated whether PSI prediction could be optimized by focusing on biologically relevant gene subsets. We applied two feature selection methods, LASSO (Least Absolute Shrinkage and Selection Operator) [22] and ASPIRE, which incorporates STG [23], to identify the most informative genes for splicing regulation. We generated distinct gene sets tailored to the task by varying the regularization coefficients for both methods. This approach allows us to dissect the biological contributions of selected genes, enhancing model interpretability and potentially uncovering novel regulators of exon-specific splicing.

**Fig 8:**
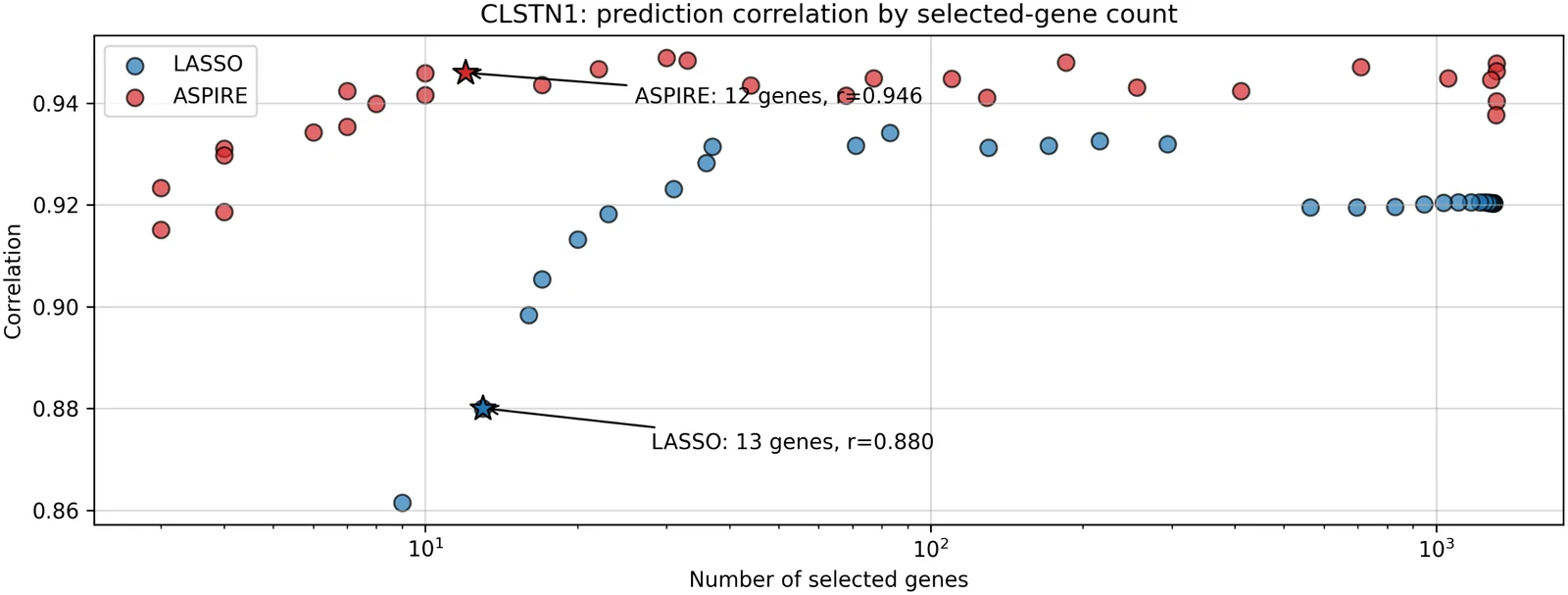
Correlation between predicted and real PSI values for LASSO and ASPIRE as a function of the number of selected genes. Correlation values are computed between $\hat{\text{y}}$ and y. Each dot represents results from training with a different regularization coefficient. Both methods exhibited a similar pattern: overfitting when a large set of genes was selected, and reduced prediction error as the number of selected genes decreased. However, ASPIRE’s predictions yield higher correlation values than LASSO for an equivalent number of selected genes. The annotated star markers indicate the minimal ASPIRE model (12 genes, r = 0.946) and the corresponding LASSO model (13 genes, r = 0.880).

ASPIRE outperformed LASSO in identifying a minimal subset of predictive genes (Fig 8). Both methods exhibited a similar pattern: overfitting when a large set of genes was selected, and a reduction in prediction error as the number of selected genes decreased. However, the predictions from ASPIRE yield higher correlation coefficients and lower MSE than those from LASSO for an equivalent number of selected genes. Notably, our results demonstrate that accurate PSI prediction can be achieved using as few as 12 genes. For the CLSTN1 exon model, the 12 ASPIRE-selected genes were: *MAP4*, *PCBP4*, *SRPK1*, *LGALS1*, *TCF20*, *SLIRP*, *ZC3H13*, *RBM47*, *ZCCHC24*, *MRPL41*, *CCDC137*, and *P4HB* (full list in S3 Table). We note that this specific set is not necessarily unique. Different optimization runs or regularization settings may yield alternative gene subsets of similar size with comparable predictive performance (see Discussion). Compared with 1,000 randomly sampled 12-gene subsets, which achieved a mean correlation of r = 0.698, the ASPIRE-selected set (r = 0.946) substantially outperformed the random baseline (S4 Fig). This reinforces that a limited yet biologically relevant gene set is sufficient to model exon-specific splicing regulation.

Our analysis demonstrates that accurate PSI prediction can be achieved using a small subset of RBP genes. The high correlation among RBPs, combined with the stochastic nature of feature selection, may lead to variability in the specific genes identified across different runs. Nonetheless, the consistent predictive power of ASPIRE highlights the robustness of this approach in leveraging biologically relevant signals for splicing analysis.

### ASPIRE demonstrates predictive robustness through uncertainty quantification with monte carlo dropout

Our model accurately predicts PSI from read coverage comparable to that typically observed in scRNA-seq. However, the noisy nature of scRNA-seq, particularly for low-expression or complex splicing patterns, could adversely affect model performance. To assess the reliability of our predictions across different splicing scenarios, we implemented Monte Carlo (MC) dropout [24], a technique for uncertainty quantification in deep learning models. MC dropout is a regularization method in which randomly selected neurons are set to zero during training and at inference. This allows for the generation of multiple stochastic predictions from the same model. By generating multiple stochastic predictions for the same input (e.g., 100 iterations in this study), we estimated predictive uncertainty by analyzing variability across these outputs.

The motivation for using MC dropout lies in its ability to provide uncertainty estimates without requiring significant changes to the model architecture. This is particularly valuable for alternative splicing analysis, where prediction confidence may vary based on factors such as tissue-specific regulation and transcript abundance. By quantifying uncertainty, we gain insight into the model’s confidence in its predictions, particularly for values at the extremes of the PSI range or for rare splicing events.

**Fig 9:**
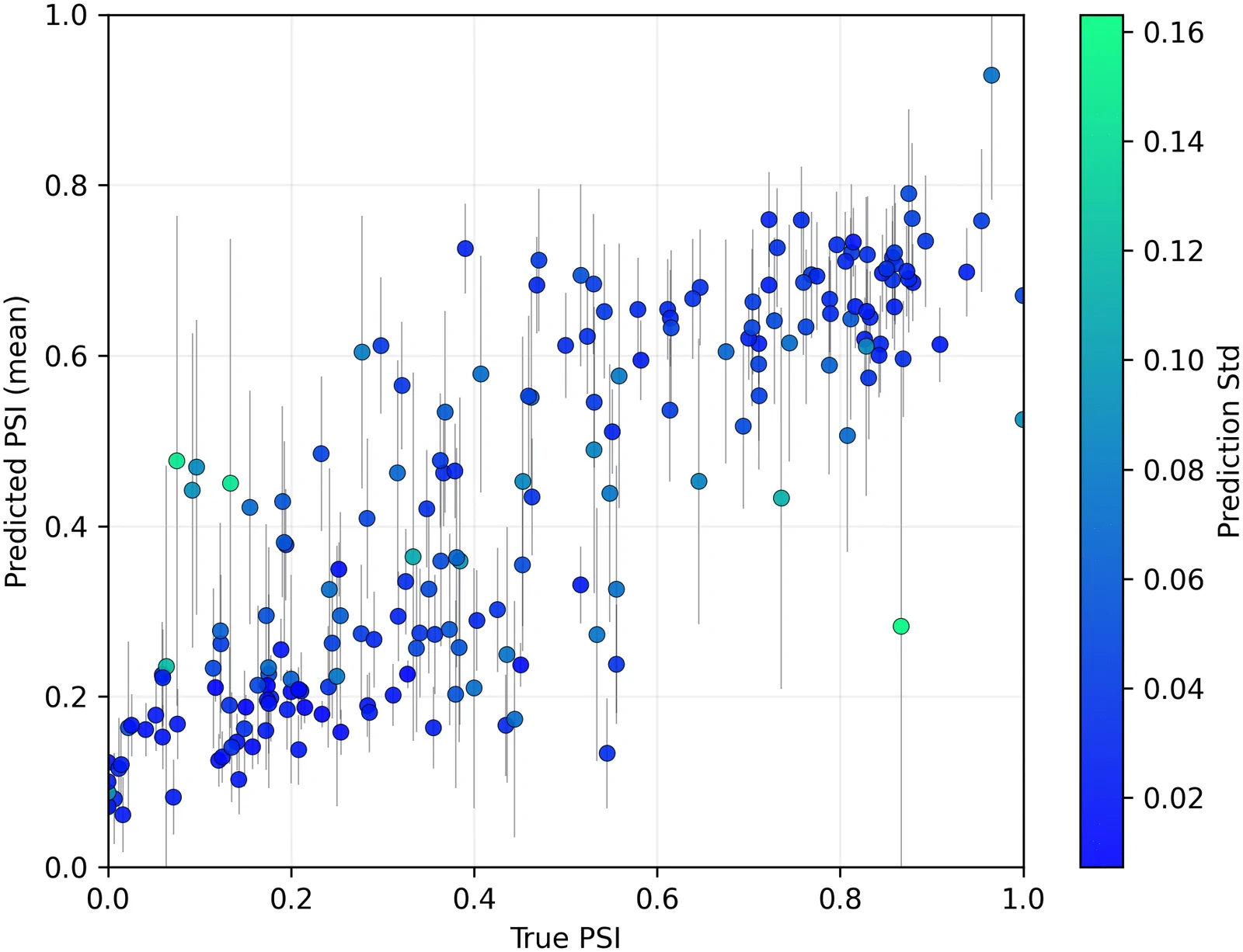
PSI Prediction Uncertainty Quantification: Multiple predictions 100 iterations (100) for the same input, with dropout enabled at inference. True PSI values are sorted in descending order and shown with their corresponding mean predictions and 95% prediction interval across all 100 runs.

In this evaluation, we trained a neural network with a 0.1 dropout rate on a subset of genes selected by ASPIRE. During inference, we introduced dropout and ran 100 stochastic forward passes. The resulting spread of predictions, as illustrated in Fig 9, highlights the robustness of the model. Results demonstrate that, despite incorporating dropout, our method consistently predicts PSI values for the chosen gene set across all PSI ranges. This robustness suggests that our selected gene set captures key regulatory relationships governing alternative splicing, even in noisy single-cell data.

To assess whether the uncertainty estimates are practically useful, we stratified predictions into five equal-count bins by their uncertainty level, measured as the standard deviation across 100 stochastic forward passes (N = 926 test predictions pooled across folds). As shown in S5 Fig, predictions with lower uncertainty were consistently more accurate: Pearson correlation with ground truth decreased from r = 0.94 in the lowest-uncertainty bin to r = 0.64 in the highest, with a corresponding increase in prediction error (RMSE from 0.12 to 0.23). Predictive uncertainty also correlated significantly with absolute error (Spearman $\rho =0.27,\;p=1.6\times 10^{-16}$), indicating that the uncertainty estimate can serve as a practical confidence filter for identifying the most reliable predictions.

### RNA-binding protein genes show better PSI prediction than other gene pools at low sequencing depths

A key motivation for this analysis is to investigate the impact of gene pool selection on PSI prediction accuracy, particularly at low sequencing depths. Different gene sets are expected to contribute differently to prediction performance, given their biological roles and expression patterns. To explore this, we compared RNA-binding protein (RBP) genes [25] against three other gene pools: alternative-splicing-relevant RBPs (82 genes curated from Tao et al. [26]), house-keeping genes [27], and random gene sets of the same size. The gene lists for all four pools are provided in S4 Table.

Housekeeping genes were chosen as the comparison group because they are consistently expressed and play a crucial role in maintaining essential cellular functions across various tissue types and conditions [27]. While their consistent expression makes them reliable for specific applications, they are not explicitly linked to splicing regulation and may lack the specificity required for accurate PSI prediction. In contrast, random gene pools serve as a baseline for evaluating the performance of RNA-binding proteins (RBPs) and housekeeping genes.

RNA-binding protein genes were hypothesized to outperform the other pools due to their direct involvement in RNA splicing and post-transcriptional regulation. RBPs are known to influence exon inclusion and alternative splicing, making them particularly relevant for predicting PSI values. By focusing on genes with mechanistic roles in splicing, we aimed to improve model performance, particularly under challenging conditions, such as low sequencing depths.

**Fig 10:**
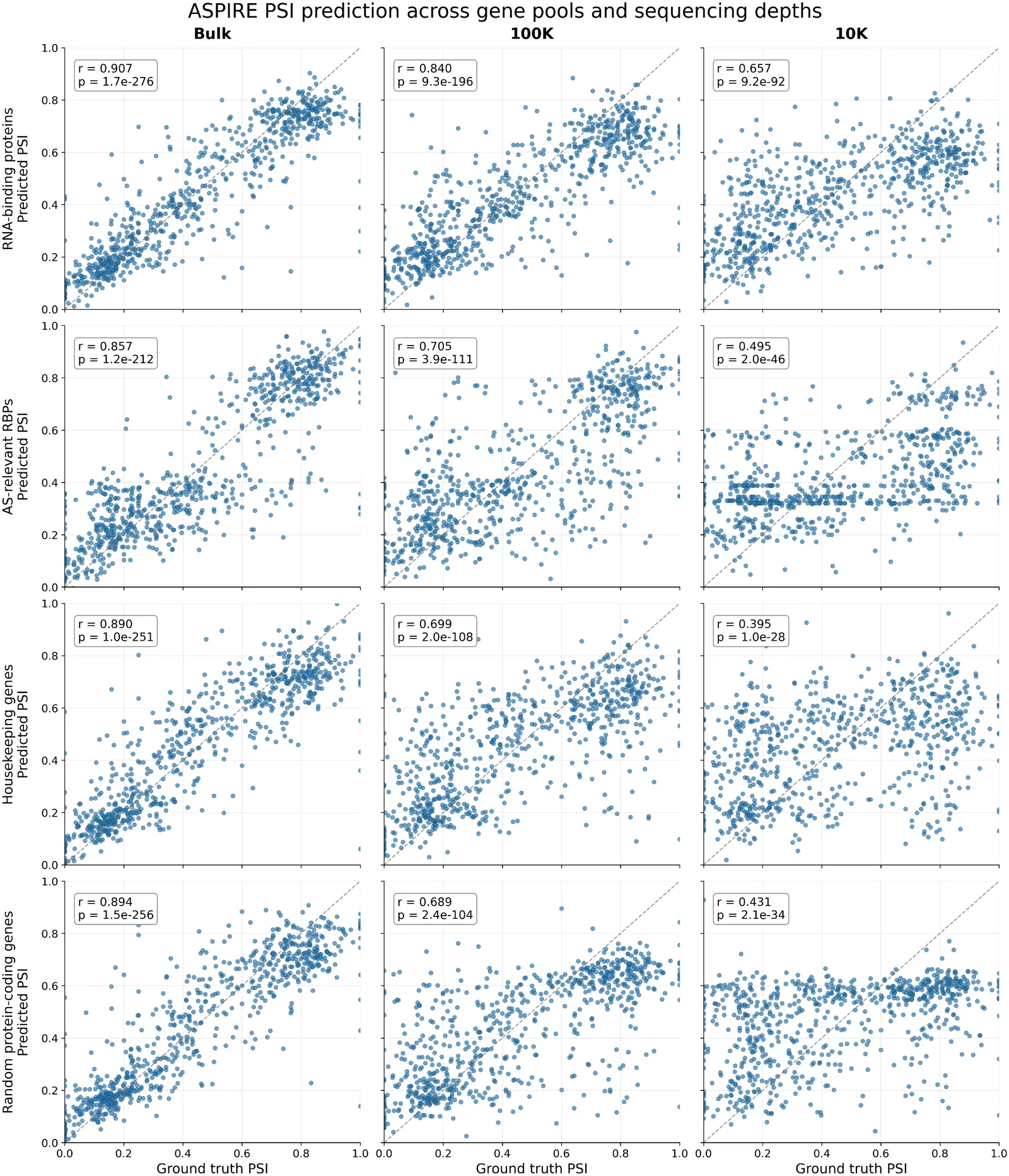
Performance comparison of PSI prediction across different gene pools at varying sequencing depths. (Bulk/100K(SC)/10K), where SC denotes single-cell, as 100K is typical of scRNA-seq depth. Each panel shows ASPIRE-predicted PSI versus ground-truth PSI, with Pearson r and two-sided p-value annotated. Rows correspond to four gene pools (from top to bottom): RNA-binding proteins (1,319 genes; average selected: 29.2 ± 1.3), AS-relevant RBPs (82 genes curated from Tao et al. 2024; average selected: 6.3 ± 0.4), housekeeping genes (average selected: 35.2 ± 4.9), and random protein-coding gene sets (average selected: 35.0 ± 3.1). Columns correspond to sequencing depths. RNA-binding protein genes consistently achieve the highest correlation across all depths. The AS-relevant RBP subset outperforms housekeeping and random sets at reduced depths (100K and 10K). Gene lists for all four pools are provided in S4 Table.

As shown in Fig 10, prediction based on RBP genes is more accurate compared with the house-keeping genes and random gene pools. The AS-relevant RBP subset showed intermediate performance at full depth (r = 0.857) but outperformed both housekeeping and random sets at reduced depths (100K: 0.705 vs 0.699/0.689; 10K: 0.495 vs 0.395/0.431), supporting the value of splicing-specific gene selection under sparse conditions. This advantage was consistently observed across different sequencing depths, including single-cell RNA sequencing (scRNA-seq) at 100,000 reads. These results underscore the importance of selecting biologically relevant gene pools for predictive tasks in RNA sequencing data.

### Evaluation of predictive robustness through selected vs. random subsets of RNA-binding protein genes

To validate the effectiveness of ASPIRE for feature selection, we conducted a comparative analysis of genes selected by ASPIRE versus randomly selected genes from the RNA-binding protein (RBP) gene pool. This analysis served two purposes: to assess whether ASPIRE identifies genuinely informative genes for PSI prediction and to demonstrate that the performance improvements aren’t simply due to the reduced feature set size.

Our evaluation methodology used ASPIRE to select a subset of predictive genes from the RBP gene pool and then generated multiple random subsets of RBP genes of the same size as the STG-selected set. We trained separate models using both the STG-selected genes and random subsets. We evaluated their predictive performance using correlation and mean squared error (MSE) between predicted and ground-truth PSI values.

**Fig 11:**
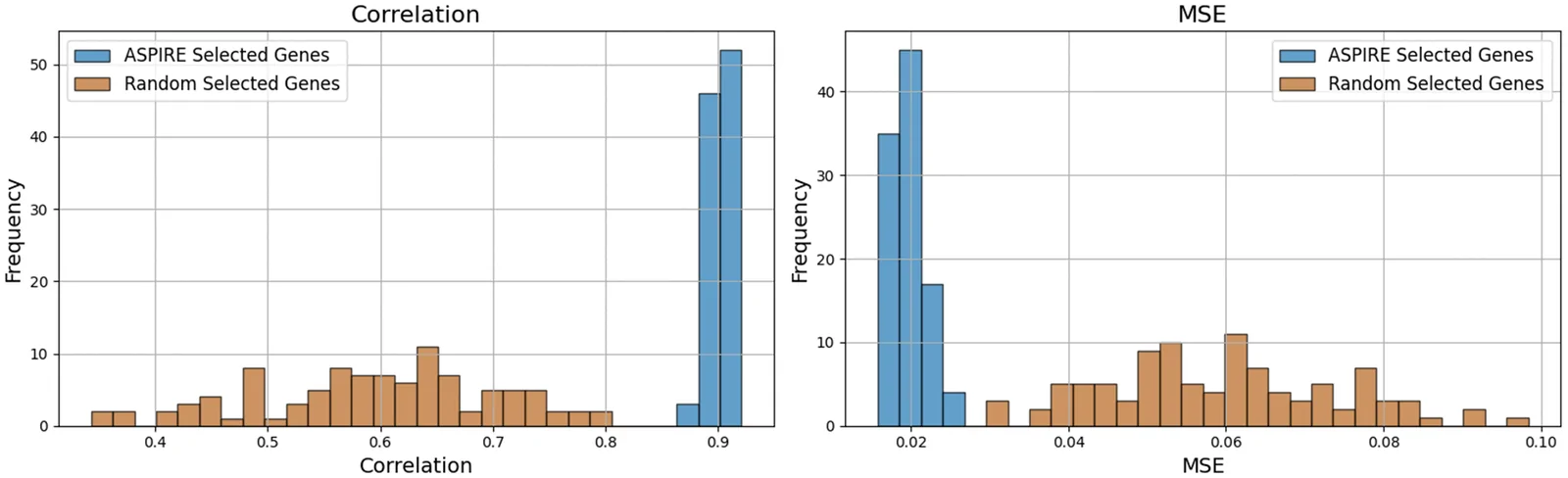
Comparison of PSI prediction performance using ASPIRE-selected genes versus random subsets from the RBP gene pool. Correlation values are computed between $\hat{\text{y}}$ and y. Results demonstrate the superiority of ASPIRE-selected genes.

As shown in Fig 11, the ASPIRE-selected genes consistently achieved higher correlation and lower MSE compared to random subsets. These results demonstrate that ASPIRE effectively identifies biologically relevant genes for PSI prediction rather than simply benefiting from a reduced feature set. The selected genes provide robust and reliable predictions, suggesting they capture fundamental relationships between gene expression and splicing regulation.

### Leave-tissue-out generalization

**Fig 12:**
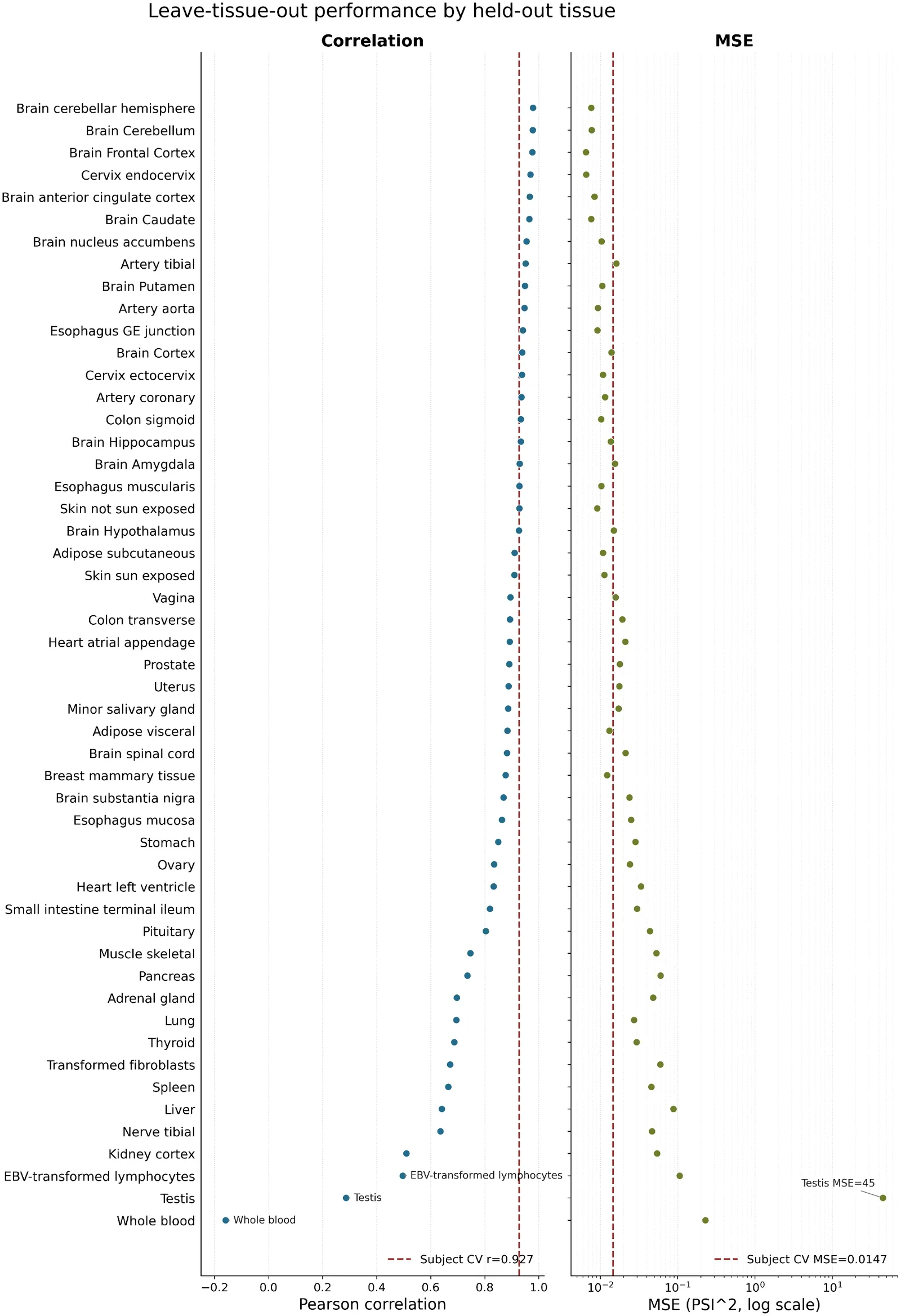
Leave-tissue-out performance by held-out GTEx tissue. ASPIRE was trained with each of the 51 GTEx tissues held out in turn and evaluated on the held-out tissue. Left panel: per-tissue Pearson correlation between predicted and ground-truth PSI. Right panel: per-tissue MSE (log scale). Dashed line indicates the subject-level cross-validation baseline. Median per-tissue correlation was 0.89 (median MSE = 0.017). Tissues are sorted by correlation in descending order.

To assess whether ASPIRE generalizes beyond tissue-specific expression patterns, we performed a leave-tissue-out cross-validation on the GTEx dataset, in which entire tissues were held out from training and used only for testing. ASPIRE retained predictive performance across most held-out tissues, with a median per-tissue Pearson correlation of 0.89 and a median MSE of 0.017 (Fig 12). Brain-related tissues achieved the highest correlations (r > 0.9), while tissues with distinct expression regimes, particularly Testis and Whole Blood, showed substantially lower performance. These results indicate that ASPIRE captures transferable regulatory signals for most tissues but remains sensitive to biologically divergent expression contexts.

### Stability of selected genes across folds and depths

**Fig 13:**
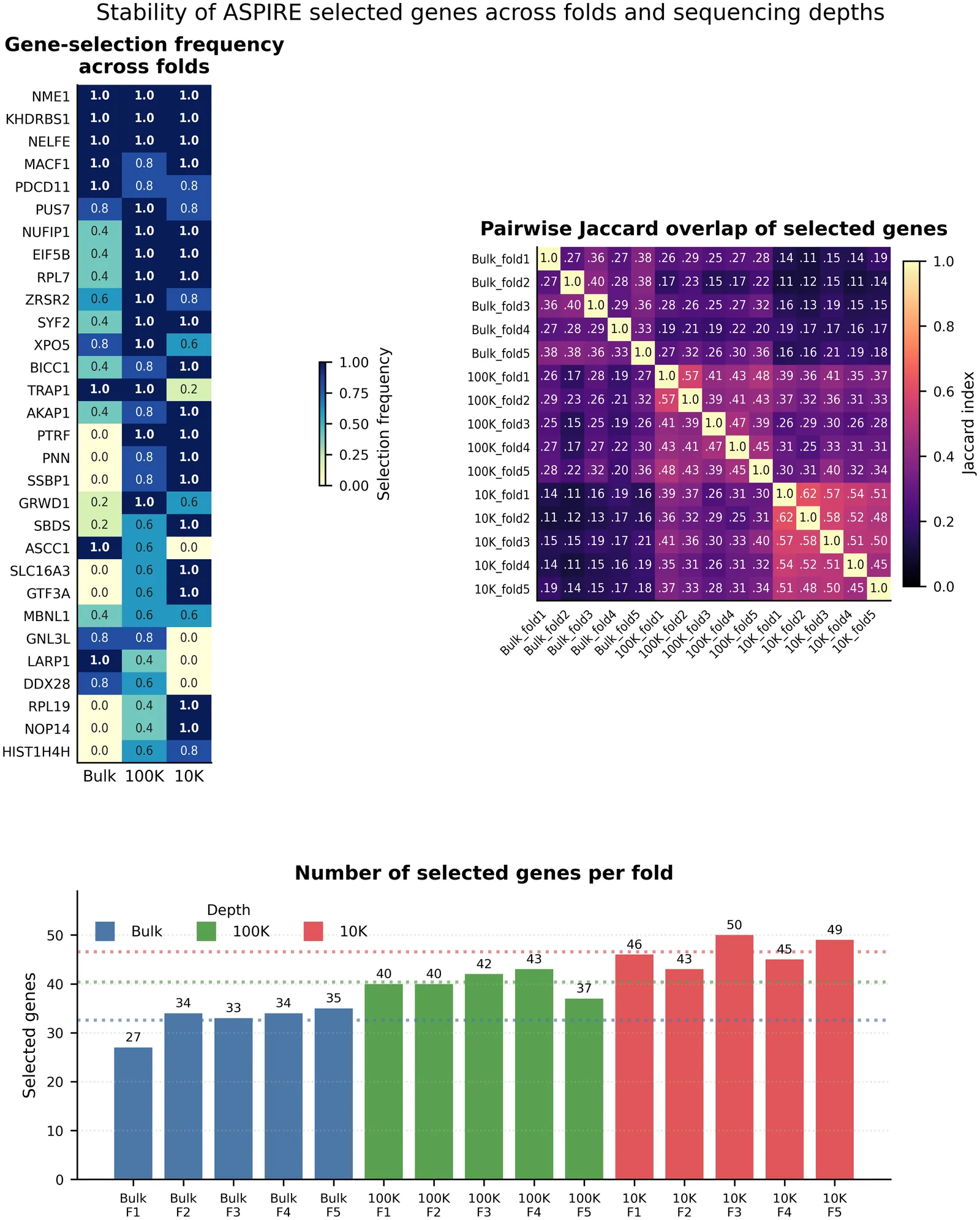
Stability of ASPIRE’s feature selection across cross-validation folds and sequencing depths. Left: selection frequency of the top recurrent genes across three sequencing depths (Bulk, 100K, 10K). A value of 1.00 indicates the gene was selected in all five folds at that depth. A core set of genes, including NME1, KHDRBS1, and NELFE, was selected consistently across all folds and depths. Right: pairwise Jaccard index of selected gene sets across all fold–depth combinations. Within-depth overlap (mean 0.434 ± 0.095) was higher than across-depth overlap (mean 0.243 ± 0.081). Bottom: number of selected genes per fold at each depth, showing a slight increase at lower depths (Bulk: 32.6 ± 3.2; 100K: 40.4 ± 2.3; 10K: 46.6 ± 2.9).

To evaluate the consistency of ASPIRE’s feature selection mechanism (Stochastic Gates, STG), we assessed gene-selection stability across five cross-validation folds and three sequencing depths (Bulk, 100K, and 10K). A core set of genes including NME1, KHDRBS1, and NELFE was selected across all folds and depths (Fig 13, left). Pairwise Jaccard overlap was higher within depth (mean 0.434 ± 0.095) than across depths (mean 0.243 ± 0.081) (Fig 13, right), indicating that while a stable core is retained, the model adapts its gene selection to the available signal at each depth. The number of selected genes was consistent across depths, averaging 32.6 ± 3.2, 40.4 ± 2.3, and 46.6 ± 2.9 genes for Bulk, 100K, and 10K, respectively (Fig 13, bottom).

### Stratified performance by PSI range and host-gene expression

**Fig 14:**
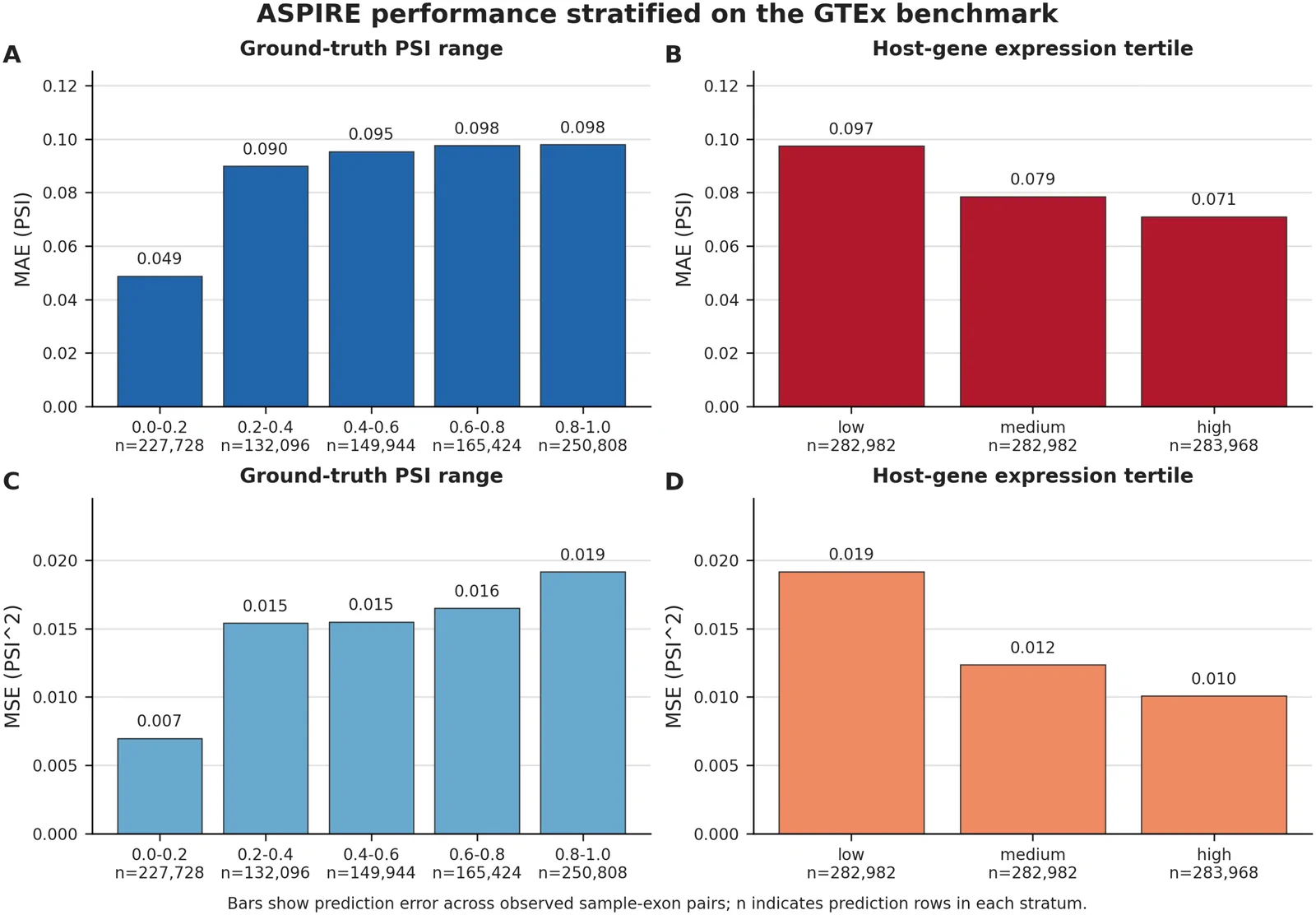
ASPIRE performance stratified on the GTEx benchmark. **(A)** MAE and **(C)** MSE stratified by ground-truth PSI range. Prediction error was lowest for low-inclusion exons (PSI 0.0–0.2: MAE = 0.049, MSE = 0.007) and broadly similar across higher ranges (MAE 0.090–0.098). **(B)** MAE and **(D)** MSE stratified by host-gene expression tertile (within-exon tertiles of $\log_{2}(\text{TPM}+1)$). Error decreased with increasing expression: MAE from 0.097 (low) to 0.071 (high), MSE from 0.019 to 0.010. Bars show prediction error computed across observed sample–exon pairs; n indicates the number of prediction rows in each stratum. Results are based on the 1,000 highest-variance cassette exons evaluated at full GTEx depth.

To characterize where ASPIRE performs best, we stratified prediction accuracy by ground-truth PSI range and by host-gene expression level on the GTEx benchmark (1,000 cassette exons). We report MAE and MSE rather than Pearson correlation, which is unreliable within narrow PSI intervals. As shown in Fig 14 (A,C), prediction error was lowest for low-inclusion exons (MAE = 0.049, MSE = 0.007 for PSI 0.0-0.2) and broadly similar across higher PSI ranges (MAE 0.090-0.098). Stratification by host-gene expression showed a clearer trend: MAE decreased from 0.097 in the low-expression tertile to 0.071 in the high-expression tertile (Fig 14 B,D), indicating that ASPIRE is most reliable for exons in highly expressed host genes.

## Discussion

This study presents ASPIRE, a deep-learning-based framework for predicting alternative splicing (AS) metrics, specifically Percent Spliced-In (PSI) values, from gene expression data obtained at low sequencing depths. ASPIRE addresses key challenges in analyzing AS in sparse single-cell RNA sequencing (scRNA-seq) datasets by leveraging transcript expression values rather than direct splice-junction coverage. Our results demonstrate that ASPIRE accurately predicts PSI values across varying sequencing depths, maintaining high predictive performance even at low read counts.

Using the GTEx dataset as a benchmark, we systematically evaluated ASPIRE’s robustness across a range of sequencing depths. We further extended this analysis by comparing ASPIRE with established single-cell splicing tools, including BRIE2 and MARVEL, on multiple independent single-cell datasets. Evaluation on a defined panel of nine biologically relevant genes demonstrated strong performance for the majority of genes across multiple data types, while also revealing that not all cassette exon events are equally predictable from expression profiles.

In these real single-cell settings, performance comparisons should be interpreted with care, as the available reference PSI values are themselves inferred from sparse single-cell data rather than derived from high-confidence bulk measurements. Consequently, the evaluation reflects agreement between different computational estimates of splicing rather than direct recovery of an absolute ground truth.

Within this context, ASPIRE demonstrated performance comparable to existing methods across many events, despite relying solely on gene expression rather than splice-junction information. This suggests that expression-based models can capture meaningful splicing-related signal even when junction-level evidence is limited, and that ASPIRE provides a complementary perspective to junction-based approaches in data regimes where direct PSI estimation is challenging.

In contrast, when reliable ground-truth PSI could be obtained from full-depth bulk RNA-seq, ASPIRE achieved substantially higher correlations with the reference PSI values. In comparison, junction-based methods struggled to provide stable or complete estimates at low sequencing depth. These results highlight a key distinction between evaluating agreement with tool-derived PSI estimates in single-cell data and evaluating recovery of biologically accurate PSI values derived from deep bulk sequencing. Together, they underscore ASPIRE’s primary strength: accurately approximating exon inclusion levels from gene expression alone when sequencing depth is insufficient for direct PSI quantification. Moreover, our comparative analysis confirmed that non-linear models, particularly ASPIRE, outperform linear approaches, reinforcing the complex and non-linear nature of splicing regulation and the need for deep-learning-based methods in low-depth transcriptomic settings. Leave-tissue-out evaluation further confirmed that ASPIRE captures transferable regulatory signals across most GTEx tissues, though performance was reduced for tissues with highly distinct expression profiles.

A key limitation of our study is that the reduced-coverage datasets used to evaluate ASPIRE were generated through random downsampling of bulk RNA-seq reads rather than from real single-cell experiments. Although this strategy effectively reproduces low read depth, a significant barrier to PSI estimation in scRNA-seq, it does not fully mimic the molecular capture variability, amplification biases, and zero-inflated expression distributions characteristic of single-cell profiling. Consequently, the 100K and 10K TPM profiles used here should be regarded as depth-matched approximations rather than precise representations of scRNA-seq data. Future work incorporating real single-cell datasets with validated splicing measurements will be crucial for further characterizing ASPIRE’s performance under true single-cell technical noise.

Feature selection was instrumental in refining PSI predictions while mitigating noise. Our findings show that restricting input features to biologically relevant subsets, particularly RNA-binding protein (RBP) genes, significantly improves predictive accuracy compared with models using housekeeping or randomly selected genes. This underscores the central role of RBPs in splicing regulation and suggests that informed gene selection strategies can markedly improve AS analyses in low-depth sequencing contexts [19,20,28]. Gene-selection stability analysis showed that a consistent core of genes was selected across cross-validation folds and sequencing depths, supporting the biological interpretability of the selected features.

Importantly, we do not present the specific gene sets identified by ASPIRE as unique or biologically optimal panels of splicing regulators. Because informative genes are often correlated and STG selection is inherently stochastic, other subsets of similar size may achieve comparable predictive performance. Rather, ASPIRE’s feature selection serves as a first-pass screen that narrows thousands of candidate genes to a compact, predictive subset, which can then be refined into a biologically meaningful panel through downstream validation or curation [29]. The central finding is that a small gene set suffices for accurate PSI prediction; identifying the most relevant regulators among these candidates is a natural follow-up to this initial filtering step.

We integrated uncertainty quantification through Monte Carlo (MC) dropout to further enhance reliability, enabling a probabilistic assessment of ASPIRE’s predictions. The consistency of confidence estimates across different PSI ranges affirms ASPIRE’s robustness, providing an additional layer of reliability for researchers analyzing low-depth transcriptomic data. We further demonstrated that low-uncertainty predictions are substantially more accurate, indicating that the MC-dropout uncertainty estimate can serve as a practical confidence filter. Stratification by PSI range and host-gene expression level revealed that ASPIRE performs best for exons in highly expressed genes.

In summary, ASPIRE constitutes a significant advancement in the computational predictions of alternative splicing, particularly in single-cell transcriptomics. By enabling accurate expression-based PSI prediction from low-depth RNA sequencing data, ASPIRE facilitates new investigations into splicing dynamics across diverse biological conditions, including disease progression, cellular heterogeneity, and therapeutic responses. This study establishes ASPIRE as a valuable tool to mitigate the limitations of scRNA-seq in AS analysis, highlighting the critical role of biologically informed machine-learning approaches in transcriptomic research.

This positioning is consistent with recent perspectives on AI-based splicing prediction, which emphasize quantitative and context-specific splicing inference while highlighting persistent challenges in cell-type-specific regulation, trans-acting regulatory effects, and multimodal integration [30].

Future work will expand ASPIRE’s capabilities by integrating multi-omic datasets, including chromatin accessibility and proteomics, to improve predictions of splicing regulation [28,30]. Additionally, ASPIRE could be adapted to predict splicing factor binding sites, facilitating its application in perturbation-based RNA-seq experiments to explore dynamic splicing responses under specific conditions. Given the interplay between alternative splicing and RNA editing, ASPIRE could also be extended to study RNA editing events and their potential impact on splicing regulation. Such extensions would further broaden ASPIRE’s utility in dissecting the regulatory mechanisms underlying transcriptomic diversity and post-transcriptional modifications.

## Methods

### Ethics statement

This study exclusively used previously published publicly available datasets for which ethical approval and informed consent were obtained by the original studies. No new human samples, animal experiments, or personally identifiable data were collected. Access to the GTEx controlled-access data was obtained through dbGaP in compliance with the GTEx data use agreement. All other datasets used are publicly available without restriction.

### Study design and validation strategy

To address the limitations of scRNA-seq in AS analysis, we propose ASPIRE (**A**ccurate **S**plicing **P**rediction from L**I**mited **R**NA S**E**quencing), a novel deep-learning approach that predicts AS metrics, specifically Percent Spliced In (PSI), using gene expressions derived from a limited number of reads equivalent to the sparse reads from scRNA-seq data. ASPIRE can effectively predict PSI from read coverage comparable to that typically found in single-cell sequencing experiments (100K). Moreover, by integrating an embedded feature selection mechanism, we identified a small, highly predictive subset of genes, enabling efficient and cost-effective analysis.

Results show that ASPIRE accurately predicts PSI values across varying sequencing depths, outperforming benchmark methods as sequencing depth drops. By leveraging Transcripts Per Million (TPM) values, we mitigate the challenges of obtaining raw transcript counts while maintaining predictive reliability. Additionally, by focusing on a minimal yet biologically relevant subset of genes, we enhance the clarity of our results and attenuate the impact of nuisance genes that can obscure meaningful biological signals.

As expected, we demonstrated that RNA-binding protein (RBP) genes play a crucial role in PSI prediction, outperforming housekeeping and randomly selected gene pools, particularly at low sequencing depth. This reinforces the biological significance of RBPs in splicing regulation and highlights their predictive value in sparse RNA-seq data. Our results showed that ASPIRE effectively maintained high correlation values when predicting PSI using RBP genes, even at sequencing depths typical of scRNA-seq. RBP-based predictions exhibited lower mean squared error (MSE) and greater robustness than other gene pools, particularly under limited read coverage.

Additionally, we performed uncertainty quantification using Monte Carlo (MC) dropout to confirm the reliability of ASPIRE’s predictions. We assessed predictive confidence across PSI ranges by introducing stochastic dropout during inference, demonstrating that ASPIRE remains robust even under noisy-data conditions.

These findings underscore the importance of gene selection strategies in splicing analysis and further validate ASPIRE as a powerful tool for studying alternative splicing across diverse biological conditions. By enabling accurate PSI predictions at low sequencing depths, ASPIRE opens new avenues for transcriptomic research, particularly in single-cell studies where read sparsity remains a major challenge.

This paper details the development and validation of ASPIRE. To rigorously assess our model, we employed a two-tiered validation strategy. First, we utilized the GTEx dataset to construct ’pseudo-single-cell’ profiles by downsampling bulk RNA-seq data. This controlled environment allows us to use PSI values derived from full-depth bulk sequencing as a high-confidence ’Gold Standard’ ground truth. Second, to demonstrate applicability in real-world scenarios and agreement with existing methods, we benchmarked ASPIRE against existing tools (BRIE2 [16] and MARVEL [17]) using independent, experimentally derived single-cell RNA-seq datasets.

ASPIRE opens new avenues for understanding splicing regulation across diverse biological contexts by enabling accurate expression-based PSI prediction with limited read depth. ASPIRE’s ability to function with sparse data makes it particularly valuable for studying cellular heterogeneity in development, disease progression, and treatment response. This advancement is a significant step toward understanding the complex role of alternative splicing in cellular functions and disease mechanisms.

## Materials and methods

This section outlines the datasets and preprocessing steps, followed by a discussion of existing methods and our proposed deep learning architecture.

### Datasets overview

To ensure robust evaluation, we utilized three distinct data sources. The Genotype-Tissue Expression (GTEx) project served as our primary dataset for model training and ’Gold Standard’ evaluation, utilizing downsampling to mimic single-cell depths while retaining bulk-derived ground truth. Additionally, we employed two independent real-world scRNA-seq datasets - Mouse Multiple Sclerosis (BRIE2 dataset) and Human hiPSC Differentiation (MARVEL dataset) - to benchmark performance against state-of-the-art tools under realistic experimental conditions.

### Primary training and evaluation data: GTEx v7

We used the Genotype-Tissue Expression (GTEx) version 7 project [31], a widely used genomic resource comprising approximately 11,700 RNA-seq samples derived from 54 tissue sites across hundreds of patients. We specifically acquired RNA-seq data from hundreds of antemortem samples to mitigate bias across 51 tissues. All participants’ demographic and clinical characteristics are detailed in S5 Table.

**Fig 15:**
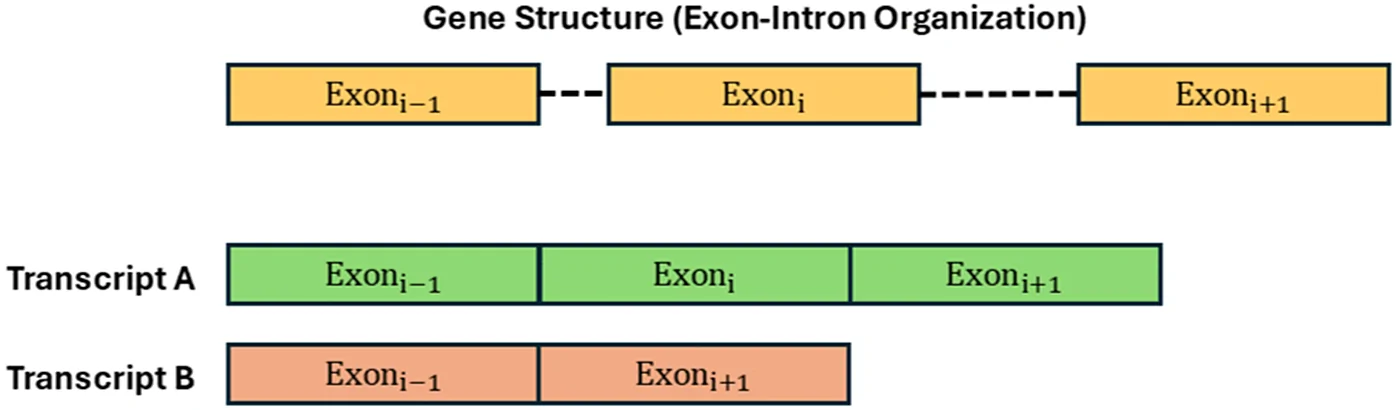
Cassette exon (exon skipping) event in alternative splicing. The top panel shows the gene structure with three exons (yellow) separated by introns (dashed lines). The bottom panel illustrates two possible mature mRNA isoforms: one that includes exon i (green) and one in which exon i is skipped, resulting in direct splicing of exon i–1 to exon i + 1. Such an alternatively included exon is referred to as a cassette exon.

For each RNA-seq sample, we computed Percent Spliced-In (PSI), a widely used metric for quantifying exon inclusion levels in alternative splicing events [32,33]. PSI is defined as:


$$\begin{array}{c} \text{PSI}=\frac{I}{I+S}\times 100, \end{array}$$ (1)

where *I* represents the number of sequencing reads that indicate the exon is *included* in the transcript, and *S* denotes the number of sequencing reads that show the exon is *skipped*. This metric provides a percentage-based estimate of exon inclusion. In this study, we specifically focus on *cassette exons* (Fig 15). To calculate PSI values, we use Equation 1 on the provided junction table from the GTEx portal.

Gene expression levels were obtained from the GTEx portal and are presented as Transcripts Per Million (TPM).

### Construction of pseudo–single-cell TPM datasets (500K, 100K and 10K)

To evaluate ASPIRE under sequencing depths characteristic of single-cell RNA-seq, we generated reduced-coverage versions of selected GTEx bulk RNA-seq libraries via systematic downsampling at the FASTQ level. For a fixed set of GTEx donors and tissues (S5 Table), we subsampled reads without replacement to fixed totals of 500,000, 100,000 and 10,000 reads per sample using ’seqtk sample’. These subsampled libraries were quantified with Salmon [34] using the same reference as the full-depth data. We refer to these as pseudo–single-cell conditions: they approximate extreme sparsity but utilize the PSI estimates from the corresponding full-depth bulk RNA-seq as a stable ground-truth reference.

While subsampling bulk RNA-seq provides a controlled way to mimic reduced sequencing depth, it does not fully reproduce unique technical properties of true single-cell RNA-seq, such as molecule capture inefficiency and zero-inflated distributions. To address this, we supplemented our evaluation with the independent single-cell datasets described below.

## External benchmark datasets

### Mouse Multiple Sclerosis (BRIE2 dataset)

We obtained the multiple sclerosis mouse single-cell RNA-seq dataset (GSE113973) analyzed in the BRIE2 study [16]. BRIE2-derived PSI values, provided in the original publication, were used as a reference for benchmarking comparisons involving this dataset, enabling assessment of concordance between ASPIRE and BRIE2. Raw FASTQ files were processed using *fastp* [35] for quality control and trimming, followed by alignment to the mm10 reference genome (GENCODE M18 annotation) using STAR [36]. The resulting BAM files were used as input to MARVEL (v2.0.5) [17], following the plate-based analysis tutorial (https://wenweixiong.github.io/MARVEL_Plate.html). Splice junction files were merged across samples, and splicing event nomenclatures were generated from rMATS [37] output and converted to MARVEL format. PSI values were computed using MARVEL’s ComputePSI function for skipped exon (SE) events with a minimum junction coverage threshold of 10 reads and an uneven coverage multiplier of 10, enabling direct comparisons among MARVEL, BRIE2, and ASPIRE. The same pipeline was applied to the GTEx downsampled datasets (500K, 100K, and 10K reads), using identical parameters. Gene expression levels were quantified with Salmon [34], and the resulting TPM matrix served as the input for ASPIRE’s PSI prediction model.

### Human hiPSC differentiation (MARVEL dataset)

We next evaluated ASPIRE on the human single-cell RNA-seq dataset profiling hiPSC differentiation toward definitive endoderm (PRJEB15062), initially analyzed in the MARVEL publication [17]. The PSI values reported by MARVEL [17] were extracted from the supplementary materials and used as a reference for comparison, allowing evaluation of agreement between ASPIRE and MARVEL. FASTQ files were processed with *fastp* [35] and aligned to the hg38 genome (GENCODE v28 annotation) using STAR [36]. The aligned BAM files were used to run BRIE2 (both *brie-count* and *brie-quant*) [16]. Gene expression values were quantified with Salmon [34], and the TPM table was used as input to ASPIRE.

### Existing splicing analysis prediction methods

Low coverage in scRNA-seq poses significant challenges for PSI estimation, as many splicing events may be missed due to insufficient read depth. Various methods have been developed to analyze alternative splicing, predict splice-site usage, and estimate PSI values [38–47].

DeepPSI [14] represents a significant advancement in leveraging deep learning for PSI prediction by incorporating gene expression data. A notable feature of this approach is its use of a learned lower-dimensional embedding layer to address the complexity of inputs from numerous genes across diverse contexts. However, DeepPSI’s reliance on substantial read depth and the absence of an active feature-selection mechanism can pose challenges, particularly for scRNA-seq data, where read-depth constraints are typical. Building on the foundational ideas introduced by DeepPSI, the method proposed here addresses these limitations. Furthermore, our method yields higher-resolution PSI estimates by employing a regression-based approach rather than a classification strategy that categorizes PSI into discrete groups.

Another tool, Single Cell Analysis of Transcript Splicing (SCATS) [15], is designed to detect differential alternative splicing events using scRNA-seq data. It employs an exon-grouping strategy with or without unique molecular identifiers (UMIs). Despite its utility, SCATS does not model nonlinear relationships between features and lacks feature-selection capabilities.

MARVEL [17], an integrated platform for alternative splicing analysis in single-cell RNA-seq data, facilitates the characterization of splicing landscapes at single-cell resolution. It is compatible with plate- and droplet-based sequencing methods and supports analyses of gene expression and splicing. However, MARVEL predicts PSI values for individual exons based on rMATS [37] output, a tool designed to detect differential alternative splicing from replicate RNA-Seq data. Many exons may receive NA values, resulting in sparse data that could limit their ability to capture comprehensive splicing patterns. Additionally, it primarily focuses on pairwise comparisons of splicing across cell types, which may not accommodate studies that require broader comparisons. Like SCATS, MARVEL lacks advanced feature selection mechanisms. It does not account for nonlinear relationships among features, which may affect its accuracy in estimating PSI values in sparse or complex datasets. These limitations underscore the need for approaches, such as the one proposed in this study, that improve PSI prediction under challenging conditions, including low read coverage and sparse data.

Specialized tools such as BRIE [16] and Outrigger [18] have significantly advanced PSI estimation directly from scRNA-seq data. BRIE uses a Bayesian hierarchical model that integrates RNA-seq data with prior biological knowledge to estimate splicing isoform ratios. In contrast, Outrigger uses an innovative splice graph approach to infer PSI values from junction reads. While Outrigger has been successfully applied to scRNA-seq data, its performance is influenced by the inherent challenges of scRNA-seq, such as low molecular capture rates, uneven transcript coverage, and difficulties in capturing alternative splicing events closer to the 5’ ends of transcripts. These limitations can result in the under-sampling of minor isoforms or events in low-abundance genes. The method proposed here builds on these insights. It demonstrates improved PSI prediction performance under scRNA-seq constraints, particularly in limited-read-coverage and sparse-data settings, making it a valuable addition to the toolbox for analyzing single-cell splicing profiles.

A deep neural network model, SpliceAI [38], has been developed to predict splice junctions from pre-mRNA sequences. While SpliceAI accurately predicts splice sites, it does not account for PSI quantification. This limitation reduces its ability to directly predict metrics related to alternative splicing.

Our approach (ASPIRE) differs from existing methods by leveraging deep learning to accurately predict PSI from low-depth sequencing data typical of scRNA-seq. It maintains robust predictions by optimizing its model for low-depth sequencing conditions. Additionally, ASPIRE incorporates an embedded feature-selection mechanism that enables it to focus on a minimal yet highly predictive subset of genes, thereby reducing computational complexity and enhancing interpretability. Furthermore, unlike methods that rely on exon-junction reads, ASPIRE bypasses these constraints by using gene expression values, making it a scalable, cost-effective solution for AS analysis in single-cell transcriptomics.

### Problem setup and proposed model architecture

Our primary objective is to develop ASPIRE, a deep learning model explicitly designed for robust PSI prediction that addresses the unique challenges posed by scRNA-seq data. These challenges include the sparsity and high dimensionality of gene expression data, as well as the typically low sequencing depth. Additionally, many genes are nuisance variables, introducing irrelevant or misleading signals that obscure biologically meaningful patterns. This can hinder model performance and complicate downstream analyses [48]. To mitigate these issues, we integrate feature selection into our model to identify a subset of relevant genes that predict PSI, thereby reducing the influence of nuisance genes while improving overall performance and interpretability.

Feature selection plays a crucial role in both machine learning and statistical analysis by identifying a subset of relevant features. This approach offers multiple benefits, including reduced experimental costs, improved model interpretability, increased computational efficiency, and better generalization to unseen data [29,49,50]. Feature selection techniques are commonly categorized into filter methods [51–56], wrapper methods [57–61], and embedded methods [62–64].

A widely used embedded method is LASSO (Least Absolute Shrinkage and Selection Operator) [22], which minimizes the loss while enforcing an $\ell_{1}$ regularization constraint on the learned feature weights:


$$\begin{array}{c} \min_{\theta }\frac{1}{N}\sum_{i=1}^{N}(\theta^{T}x_{i}-y_{i}{)}^{2}+\lambda \|\theta {\|}_{1}. \end{array}$$

However, LASSO can shrink model parameters excessively and fail to capture nonlinear relationships among features. Additionally, LASSO selects features based on feature weights, which may not always align with their actual predictive importance, particularly in high-dimensional, noisy datasets such as scRNA-seq. To address these limitations, [23] proposed Stochastic Gates (STG), an embedded feature selection method that approximates $\ell_{0}$ regularization using a probabilistic relaxation. Unlike LASSO, which applies a uniform shrinkage penalty, STG enables dynamic, adaptive feature selection by learning the probability that each gene is relevant. This flexibility helps avoid excessive pruning of weak but informative genes while still eliminating irrelevant features. Each feature selection variable is modeled as a clipped Gaussian-distributed random variable:


$$\begin{array}{c} z_{d}=\max(0,\min(1,\mu_{d}+\epsilon_{d})),\;\epsilon_{d}\sim 𝒩(0,\sigma^{2}). \end{array}$$

This allows $\ell_{0}$ regularization to be approximated in a differentiable form:


$$\begin{array}{c} 𝔼[\|𝐳{\|}_{0}]=\sum_{d=1}^{D}P(z_{d}>0)=\sum_{d=1}^{D}\left(\frac{1}{2}-\frac{1}{2}\mathrm{erf}\left(-\frac{\mu_{d}}{\sqrt{2}\sigma }\right)\right), \end{array}$$

where erf(·) is the Gaussian error function.

Our model, ASPIRE, integrates STG-based regularization for PSI prediction. It consists of a multi-layer, fully connected neural network that incorporates stochastic gates in its input layer for gene selection. The prediction of the model is denoted as: $f_{\theta }({\text{x}}_{i}⊙\text{z})={\hat{\text{y}}}_{i},$ where ${\text{x}}_{i}\in {\mathbb{R}}^{g}$ represents the Transcripts Per Million (TPM) profile of the *i*-th sample, $\text{z}\in \{0,1{\}}^{g}$ are stochastic gates controlling feature selection, and ${\hat{\text{y}}}_{i}\in {\mathbb{R}}^{e}$ is the predicted PSI vector.

The training objective combines Mean Squared Error (MSE) loss with STG-based regularization:


$$\begin{array}{c} ℒ=𝔼[\frac{1}{N}\sum_{i=1}^{N}\|{\text{y}}_{i}-f_{\theta }({\text{x}}_{i}⊙\text{z}){\|}_{2}^{2}+\lambda \|𝐳{\|}_{0}]. \end{array}$$

The first term ensures accurate PSI prediction, while the second promotes feature selection. Unlike $\ell_{1}$ regularization, which uniformly shrinks all feature weights, this approach selectively prunes irrelevant genes by pushing their gate parameters $\mu_{d}$ toward negative values, ensuring that only the most informative genes contribute to the prediction.

## Supporting information

S1 Table Gene panel: cassette exon coordinates genome references. Cassette exon coordinates in human (hg38) and mouse (mm10) for the nine-gene evaluation panel (CLSTN1, PTBP1, PTBP2, ADD3, MAPT, APP, CAMK2D, BIN1, NUMB).
(XLSX)

S2 Table Per-exon correlation results for the gene panel. Pearson correlation coefficients, p-values, MSE, and sample counts for all exon-dataset-method combinations evaluated in the gene panel analysis.
(CSV)

S3 Table Selected genes for the CLSTN1 exon model. List of genes selected by ASPIRE’s Stochastic Gates (STG; 12 genes) and by LASSO (13 genes) for prediction of PSI values for exon 3 of the CLSTN1 gene (chr1:9756481–9756510, GRCh38).
(XLSX)

S4 Table Gene pools used for PSI prediction comparison. Gene lists for the four gene pools compared in Fig 10: RNA-binding proteins, AS-relevant RBPs (curated from Tao et al. [26]), housekeeping genes, and random protein-coding gene sets.
(XLSX)

S5 Table Clinical characteristics of all study participants. Summarizes the clinical characteristics of all study participants. The data, extracted from the Genotype-Tissue Expression (GTEx) project (version 7), include RNA-seq samples collected across 51 tissue types. These data supported appropriate cohort selection and helped mitigate potential biases due to biological variability among samples.
(XLSX)

S1 FigBRIE2-derived PSI and ASPIRE-predicted PSI values for representative exons.The three subplots (left to right) correspond to the top three exons with the highest correlation between ASPIRE predictions and BRIE2-derived PSI values: ENSMUSG00000027012.AS2 (correlation = 0.7901, MSE = 0.0005), ENSMUSG00000022892.AS2 (correlation = 0.7539, MSE = 0.0506), and ENSMUSG00000022892.AS3 (correlation = 0.6156, MSE = 0.0362). Correlation and MSE values are computed between $\hat{\text{y}}$ and y.(TIF)

S2 FigMARVEL-derived PSI and predicted PSI values from ASPIRE and BRIE2.The two subplots illustrate representative exons in which either ASPIRE or BRIE2 achieves superior agreement with the MARVEL-derived reference. Left: exon chr1:45606482–45606591, where ASPIRE outperforms BRIE2 (ASPIRE: *r* = 0.447, MSE = 0.0648; BRIE2: r=−0.663, MSE = 0.3010). Right: exon chr5:80637883–80638009, where BRIE2 outperforms ASPIRE (BRIE2: *r* = 0.709, MSE = 0.0005; ASPIRE: *r* = 0.064, MSE = 0.0366). Correlation and MSE values are computed between predicted PSI values $\hat{\text{y}}$ and MARVEL-derived reference PSI values y.(TIF)

S3 Fig PSI prediction correlation heatmap - all exons. Same layout and color scale as Fig 6, but showing all individual cassette exons rather than one per gene. Rows are grouped by gene and sorted within each gene by descending ASPIRE correlation at full GTEx depth. Exons with no computable correlation in any dataset are excluded.
(TIF)

S4 Fig Random 12-gene baseline for the CLSTN1 exon model. Distribution of held-out Pearson correlations from 1,000 randomly sampled 12-gene subsets (grey histogram), compared with the ASPIRE-selected 12-gene set (red vertical line, r = 0.946) and the LASSO-selected 13-gene set (blue vertical line, r = 0.880). Random subsets achieved a mean correlation of r = 0.698; none reached the performance of the ASPIRE-selected set.
(TIF)

S5 FigReliability of ASPIRE predictions as a function of Monte Carlo dropout uncertainty.Predictions (N = 926 test samples pooled across folds) were stratified into five equal-count bins by their uncertainty, measured as the standard deviation across 100 stochastic forward passes. Left: Pearson correlation between predicted and ground-truth PSI decreases monotonically from r = 0.94 in the lowest-uncertainty bin to r = 0.64 in the highest. Right: prediction error (RMSE and MAE) increases correspondingly from 0.12 to 0.23 (RMSE). Predictive uncertainty correlated significantly with absolute error (Spearman $\rho =0.27,\;p=1.6\times 10^{-16}$).(TIF)
