# Supplementary material for: ASPIRE: Accurate alternative splicing prediction from limited RNA sequencing data and a minimal gene set
Source: PLoS Comput Biol. 2026 Aug 28;22(8):e1014725. doi: 10.1371/journal.pcbi.1014725 (PMC13552950; doi:10.1371/journal.pcbi.1014725)
Supplement: Supplementary file 1 [file pcbi.1014725.s012.pdf]

# Response to Reviewers

**Manuscript PCOMPBIOL-D-26-00569**

*ASPIRE: Accurate alternative splicing prediction from limited RNA sequencing data and a minimal gene set*

PLOS Computational Biology

---

Dear Dr. Ewing, Dr. Libbrecht, and Reviewers,

We thank the Academic Editor, the Section Editor, and both reviewers for their careful and constructive evaluation of our manuscript. We are encouraged that the reviewers found the work to address an important problem and to be well executed. We have revised the manuscript to address every point raised. Below, we respond to each comment in turn; reviewer comments are shown in italics, followed by our response. Changes in the manuscript are highlighted in the accompanying tracked-changes file, with line numbers referenced where relevant.

## Response to the Editor

> **Editor:** *The reviewers' comments were overall positive. Please address the clarity of claims raised by reviewer 1 and address the questions regarding choices of gene sets raised by reviewer 2.*

**Response:** We have addressed all points raised by both reviewers. To address the clarity of claims (Reviewer 1), we revised the framing throughout the manuscript to explicitly state that our method, ASPIRE, predicts PSI (Percent Spliced-In) from gene-expression features rather than directly measuring splicing, and we tempered the single-cell conclusions accordingly. We also added four new analyses requested by Reviewer 1: a leave-tissue-out evaluation, a gene-selection stability analysis, a demonstration that low-uncertainty predictions are more reliable, and a stratification of performance by PSI range and host-gene expression level. As a further response to Reviewer 1's request for a clearer distinction between the two approaches to splicing measurement, we added a graphical abstract (now Fig 1, at the end of the Introduction) that contrasts conventional junction-based PSI estimation with ASPIRE's expression-based prediction. To address the gene-set questions (Reviewer 2), we annotate the selected genes and their counts, report results on a consistently defined, biologically based gene panel across datasets, add an alternative-splicing-specific RNA-binding proteins (RBPs) pool to the comparison, and display the MARVEL/BRIE2 benchmarking results as figure panels. We also incorporated several recent references to better situate our work within recent developments in the field. Full details are given in our responses to the individual comments below.

## Response to Reviewer #1

> **Reviewer #1:** *This is a strong and useful paper with a clear goal of recovering biologically meaningful PSI estimates from limited RNA-seq data, especially under low depth and single cell like conditions. The problem is important, the motivation is solid, and the authors do a good job showing that ASPIRE performs competitively across bulk, downsampled, and external single-cell-related datasets. The addition of biologically informed feature selection and uncertainty estimation is a real strength and makes the method more interesting than a purely technical predictor….Overall, this is a good and well executed study. It addresses a real need, is biologically important, and appears useful. I suggest a more cautious language and a few targeted analyses, then the paper would become stronger, clearer, and more accurate.*

We thank Reviewer #1 for the positive assessment and for the constructive suggestions. As suggested, we have adopted more cautious language throughout the manuscript regarding ASPIRE's capabilities (as described below in sections 1.1-1.2), and have conducted all the targeted analyses proposed by the reviewer (sections 1.3-1.6).

### 1.1

> **Reviewer #1:** *My main suggestion is to sharpen the claims slightly. ASPIRE does not directly measure splicing, but predicts PSI from expression features that are associated with splicing behavior. That is still valuable, but the manuscript would be stronger if this distinction were stated more clearly throughout.*

**Response:** We have revised the language throughout the manuscript to draw this distinction clearly. Across the Abstract (Lines 10-11, 20), Author Summary (Lines 33-34, 37), Introduction (Lines 79-82), Results (Lines 102-103, 18-191), Discussion (Lines 410, 460-462), and Methods (Line 527), we ensured that ASPIRE is consistently described as predicting PSI from gene-expression features rather than directly measuring or quantifying splicing. All edits are marked in the tracked-changes manuscript, for example, 'a deep learning framework for predicting alternative splicing from low-depth RNA-seq data' (line 10) was revised to 'a deep learning framework for predicting alternative splicing **metrics** from low-depth RNA-seq gene expression data'; 'By enabling robust splicing inference from sparse data' (line 20) was revised to 'By enabling robust **expression-based splicing inference** from sparse data'; 'ASPIRE accurately predicts splicing patterns' (line 33) was revised to 'ASPIRE accurately predicts **splicing metrics, such as PSI, from gene expression profiles**'. To reinforce this distinction visually, we additionally added a graphical abstract (now Fig 1, at the end of the introduction; shown below) that contrasts conventional junction-based PSI estimation, which measures splicing directly from junction reads, with ASPIRE's expression-based prediction of PSI.

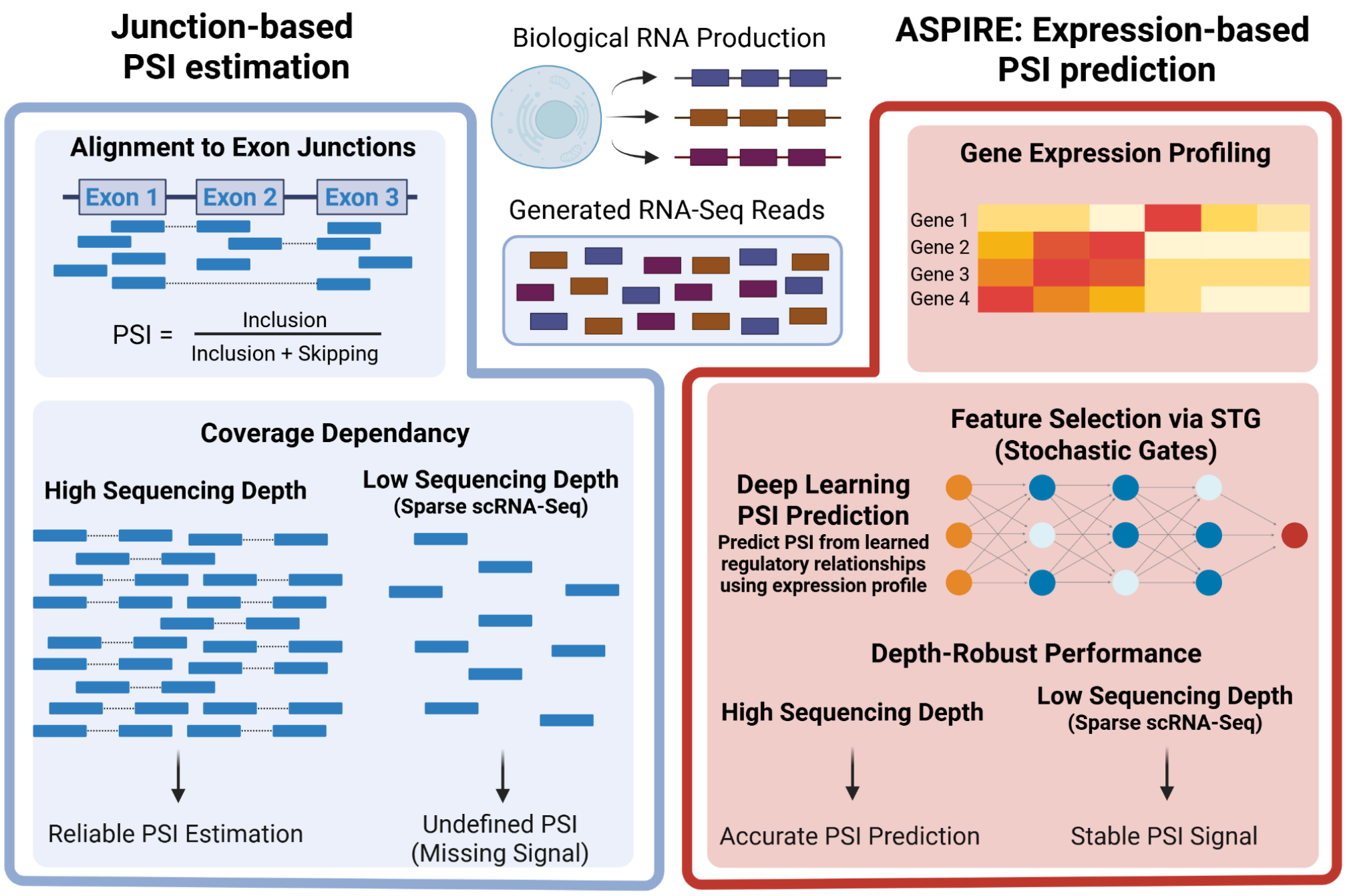


## 1.2

> **Reviewer #1:** *The single-cell validation is encouraging, but it still relies largely on agreement with reference computational methods rather than orthogonal ground truth, so the conclusions should stay careful in that regard.*

**Response:** We agree, and the existing Discussion caveat (Lines 403-407) clearly states this distinction. We have tightened the language accordingly in the relevant Results sections: in the Mouse Multiple Sclerosis (MS) results, the claim about learning expression-splicing relationships now refers to concordance with BRIE2 estimates (Lines 157-158); in the hiPSC comparison, the conclusion is framed as agreement rather than as evidence of robustness (Lines 187-192).

## 1.3

> **Reviewer #1:** *A few simple additions could strengthen the paper. A leave-tissue-out evaluation would test whether the model generalizes beyond tissue-specific expression patterns.*

**Response:** To address the reviewer's comment, we applied a Leave-tissue-out evaluation and showed that ASPIRE  retains predictive performance across most held-out GTEx tissues, with a median per-tissue correlation of 0.89 and a median Mean Squared Error (MSE) of 0.017. Fig. 12 (shown below) further illustrates the tissue-specific performance profile, showing robust generalization across most tissues, with Testis as a distinct outlier exhibiting reduced extrapolation performance. These results indicate that ASPIRE captures transferable regulatory signals across diverse tissues and highlight its expected sensitivity to highly distinct tissue-specific expression regimes. We added this analysis as a new subsection of the Results, "Leave-tissue-out generalization," beginning on line 359 of the revised manuscript.

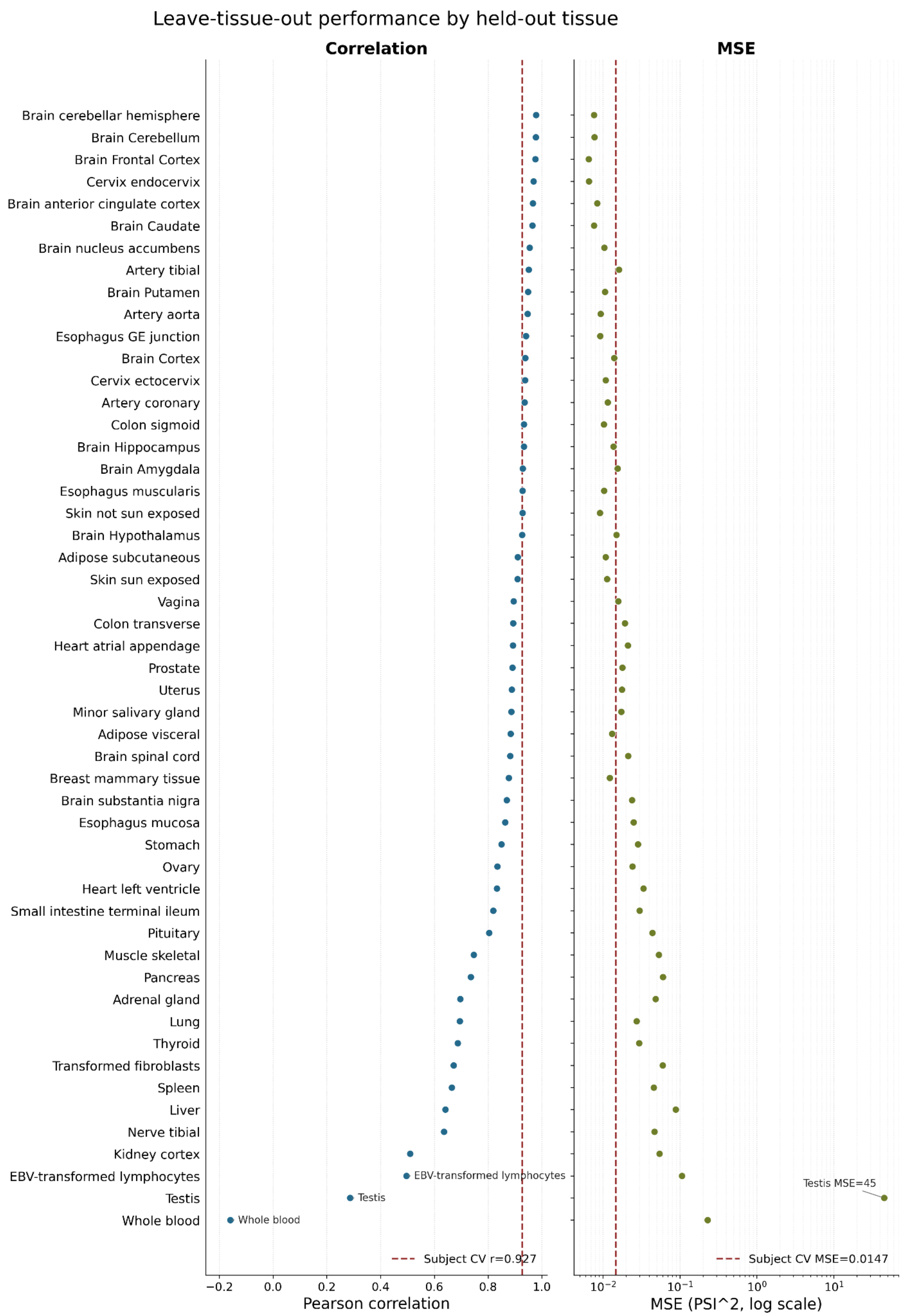

Leave-tissue-out performance by held-out tissue
Correlation
MSE
Brain cerebellar hemisphere
Brain Cerebellum
Brain Frontal Cortex
Cervix endocervix
Brain anterior cingulate cortex
Brain Caudate
Brain nucleus accumbens
Artery tibial
Brain Putamen
Artery aorta
Esophagus GE junction
Brain Cortex
Cervix ectocervix
Artery coronary
Colon sigmoid
Brain Hippocampus
Brain Amygdala
Esophagus muscularis
Skin not sun exposed
Brain Hypothalamus
Adipose subcutaneous
Skin sun exposed
Vagina
Colon transverse
Heart atrial appendage
Prostate
Uterus
Minor salivary gland
Adipose visceral
Brain spinal cord
Breast mammary tissue
Brain substantia nigra
Esophagus mucosa
Stomach
Ovary
Heart left ventricle
Small intestine terminal ileum
Pituitary
Muscle skeletal
Pancreas
Adrenal gland
Lung
Thyroid
Transformed fibroblasts
Spleen
Liver
Nerve tibial
Kidney cortex
EBV-transformed lymphocytes
Testis
Whole blood
EBV-transformed lymphocytes
Testis
Whole blood
Testis MSE=45
Subject CV r=0.927
Subject CV MSE=0.0147
−0.2
0.0
0.2
0.4
0.6
0.8
1.0
Pearson correlation
$10^{-2}$
$10^{-1}$
$10^{0}$
$10^{1}$
MSE (PSI^2, log scale)

## 1.4

> **Reviewer #1:** *A stability analysis of the selected genes across folds and depths would support the biological interpretability claims.*

**Response:** We assessed the stability of ASPIRE's feature-selection mechanism (Stochastic Gates, STG)  across five cross-validation folds and three sequencing depths by training depth-specific STG models for Bulk, 100K, and 10K inputs. The number of selected genes was consistent across depths, averaging 32.6 ± 3.2, 40.4 ± 2.3, and 46.6 ± 2.9 genes for Bulk, 100K, and 10K, respectively. Supplementary Fig. 5 (shown below) summarizes the results in three panels: (top-left) a heatmap showing the selection frequency of the top recurrent genes across depths, revealing a core set - including NME1, KHDRBS1, and NELFE - selected in all folds and depths; (top-right) a pairwise Jaccard overlap matrix showing that gene-set agreement is higher within the same depth (mean 0.434 ± 0.095) than across depths (mean 0.243 ± 0.081); and (bottom) the number of selected genes per fold, showing a slight increase at lower depths. These results support the interpretability of the STG-selected features and indicate expected changes in the selected gene sets under reduced sequencing depth. The figure was added as Fig. 13. We added this analysis as a new subsection of the Results, “Stability of selected genes across folds and depths ”, beginning on line 368 of the revised manuscript.

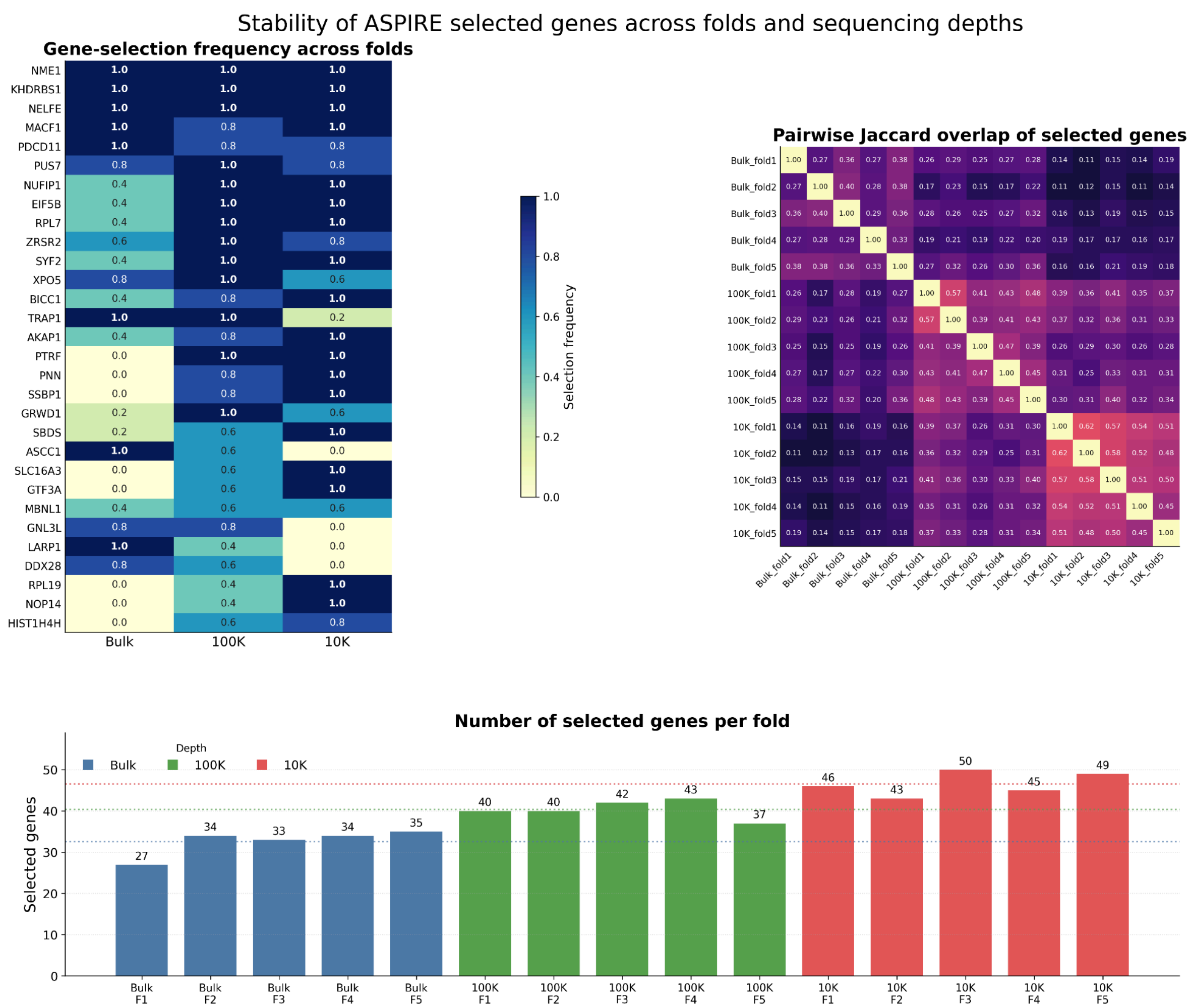

## 1.5

> **Reviewer #1:** *A short analysis showing that low-uncertainty predictions are indeed more reliable would also make the uncertainty framework more practical for users.*

**Response:** To assess whether the uncertainty estimates from Monte Carlo (MC) dropout are practically useful, we stratified predictions into five equal-count bins by their uncertainty level (measured as the standard deviation across 100 stochastic forward passes; N = 926 test predictions pooled across folds). As shown in the new Supplementary Fig. 5 (shown below), predictions with lower uncertainty were consistently more accurate: the left panel shows that Pearson correlation with ground truth decreased from r = 0.94 in the lowest-uncertainty bin to r = 0.64 in the highest, and the right panel shows a corresponding increase in prediction error (RMSE from 0.12 to 0.23). Predictive uncertainty also correlated significantly with absolute error (Spearman $\rho = 0.27$, $p = 1.6 \times 10^{-16}$). These results indicate that the uncertainty estimate can serve as a practical confidence filter for identifying the most reliable predictions. We added this analysis at the end of "ASPIRE Demonstrates Predictive Robustness Through Uncertainty Quantification with Monte Carlo Dropout" results subsection, beginning on line 305 of the revised manuscript.

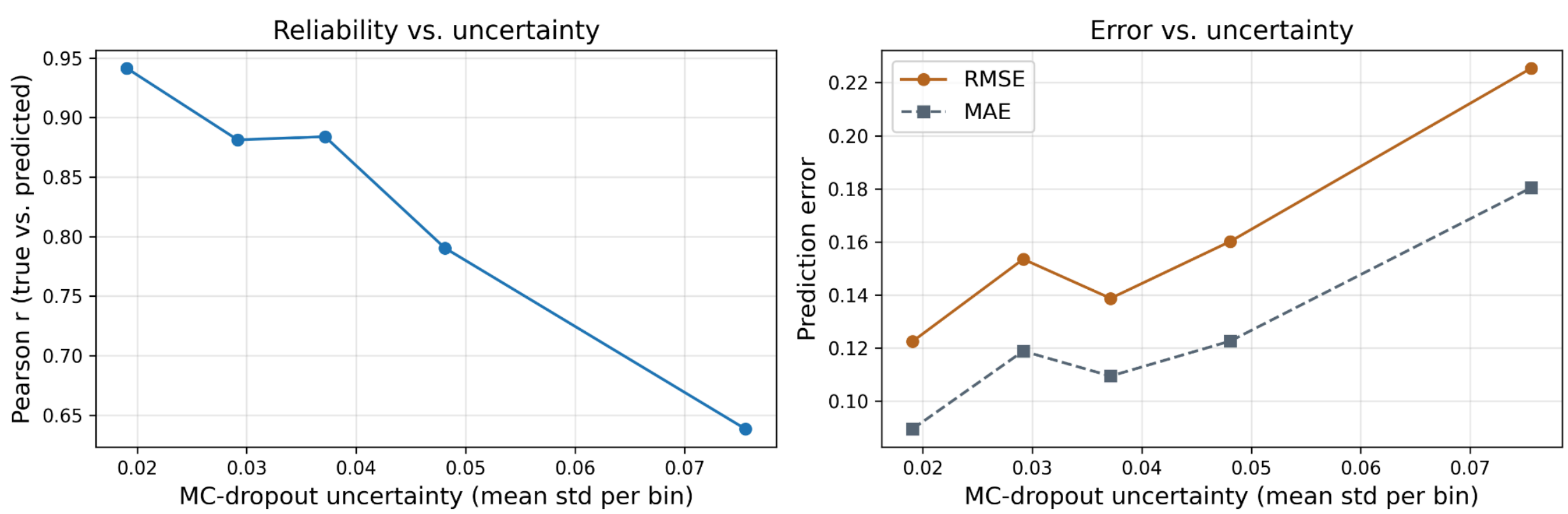


## 1.6

> **Reviewer #1:** *It would also help to stratify performance by exon type, expression level, or PSI range, to show where the model works best.*

**Response:** We thank the reviewer for this suggestion. As the current study focuses on cassette exons, we stratified ASPIRE's performance by ground-truth PSI range and by host-gene expression level on the GTEx benchmark (1,000 cassette exons). The new Fig. 14 (shown below) presents the results in four panels: panels A and C show that prediction error was lowest for low-inclusion exons (MAE = 0.049, MSE = 0.007 for PSI 0.0-0.2) and broadly similar across higher PSI ranges (MAE 0.090–0.098); panels B and D show a clearer expression-dependent trend, with MAE decreasing from 0.097 in the low-expression tertile to 0.071 in the high-expression tertile, indicating that ASPIRE is most reliable for exons in highly expressed genes. These results have been added to the manuscript. We added this analysis as a new subsection of the Results, "Stratified performance by PSI range and host-gene expression", beginning on line 377 of the revised manuscript.

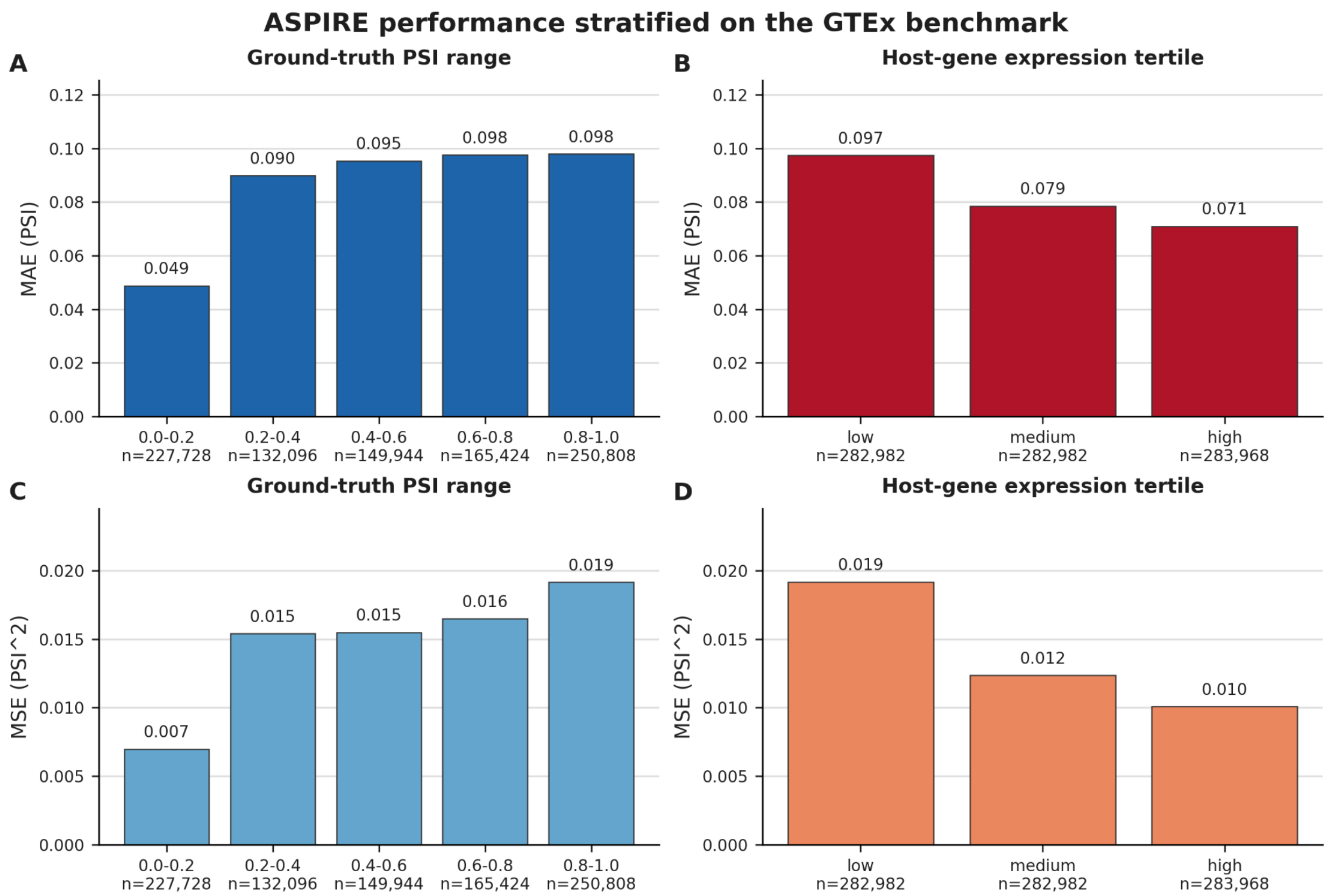


Bars show prediction error across observed sample-exon pairs; n indicates prediction rows in each stratum.

## Response to Reviewer #2

> **Reviewer #2:** *This manuscript by Eisenberg et al introduces an innovative tool called "ASPIRE", designed to predicted alternative splicing when the sequencing reads are too sparse. They benchmarked their model in both bulk-RNA-seq data and single-cell transcriptomic data, demonstrating their tool outperformed currently widely used tool such as MARVEL and BRIE2 with just expression profiles.*
>
> *However, there are some limitations regarding the evaluation dimensions and the consistence of the validation metrics. It will strengthen this study if the authors can address my comments below.*

We thank Reviewer #2 for the supportive assessment and for the detailed suggestions regarding gene-set choices and validation consistency, which have strengthened the manuscript.

### Major comment 1

> **Reviewer #2:** *Please annotate the name and the number of the selected genes in Fig5. The author claimed a minimum of 12 genes is sufficient to build the model. Please display the correlation between PSI for LASSO and ASPIRE.*
>
> *Is the 12 gene a random subset of the complete gene set?*

**Response:** We thank the reviewer for this helpful suggestion. To improve clarity while keeping the figure readable, we now report the number of selected genes in the new Fig. 8 (previously Fig. 5; top figure shown below) caption and provide the full list of selected genes in Supplementary Table 3. For the CLSTN1 exon model, ASPIRE selected 12 genes: MAP4, PCBP4, SRPK1, LGALS1, TCF20, SLIRP, ZC3H13, RBM47, ZCCHC24, MRPL41, CCDC137, and P4HB.

Notably, we do not claim these 12 genes are the unique or most important set for splicing. Other subsets of similar size likely perform comparably or better. We now frame ASPIRE's selection as a first-pass filter that narrows the candidate pool to a small predictive panel, which can be refined through biological validation or manual curation (Chandrashekar 2014, "A survey on feature selection methods"). The central message is that a small, biologically informed gene set suffices for accurate PSI prediction, its precise composition being one of several effective possibilities. We also indicate this in the Discussion part now (Lines 441-448 of the revised manuscript).

The 12-gene ASPIRE model achieved Pearson correlation r = 0.946 with the observed PSI, whereas the LASSO model with a comparable number of genes (13) achieved r = 0.880. The 12-gene set is not a random subset of the complete gene set - it was selected by ASPIRE's feature-selection mechanism (STG) from 1,319 candidate genes. To further assess this, we evaluated 1,000 randomly sampled 12-gene subsets, which achieved a mean correlation of r = 0.698 - substantially lower than the ASPIRE-selected set (Supplementary Fig. 4; bottom figure shown below). Accordingly, subsection result "ASPIRE Enables Accurate Prediction of Exon PSI Using Only a Small Gene Set" was updated (Lines 267-274 of the revised manuscript).

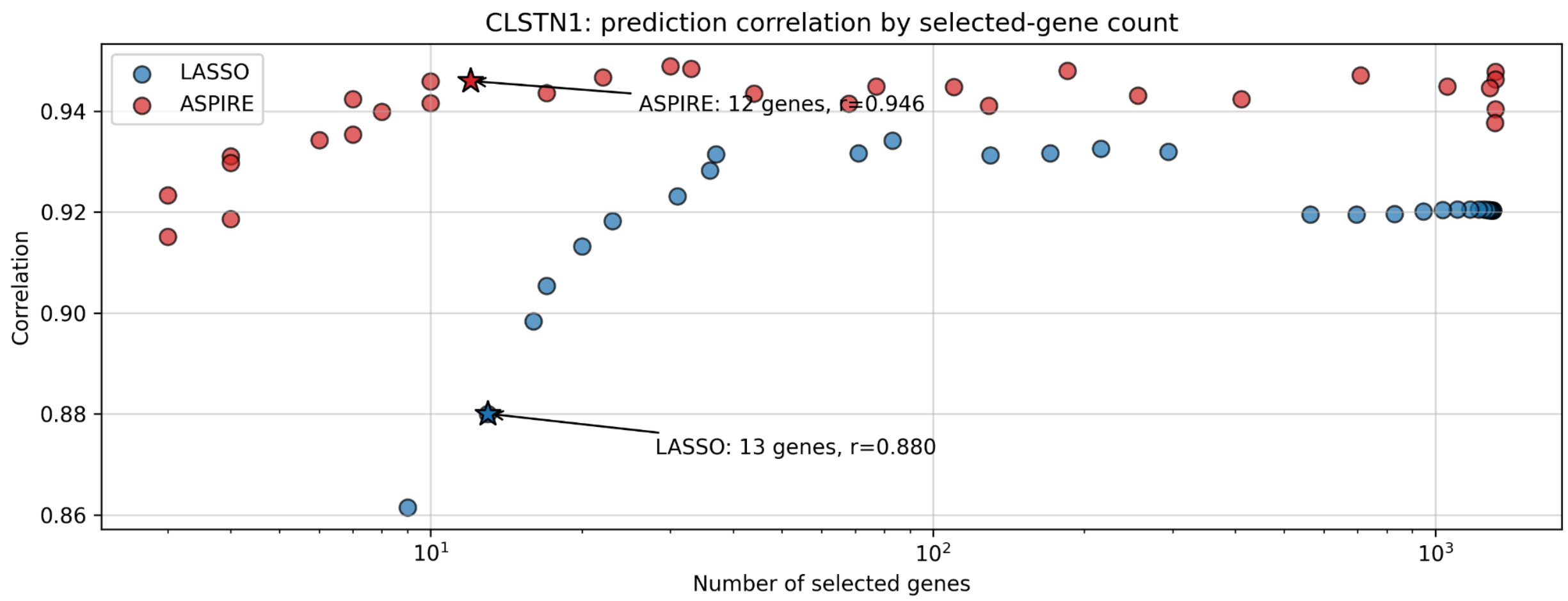


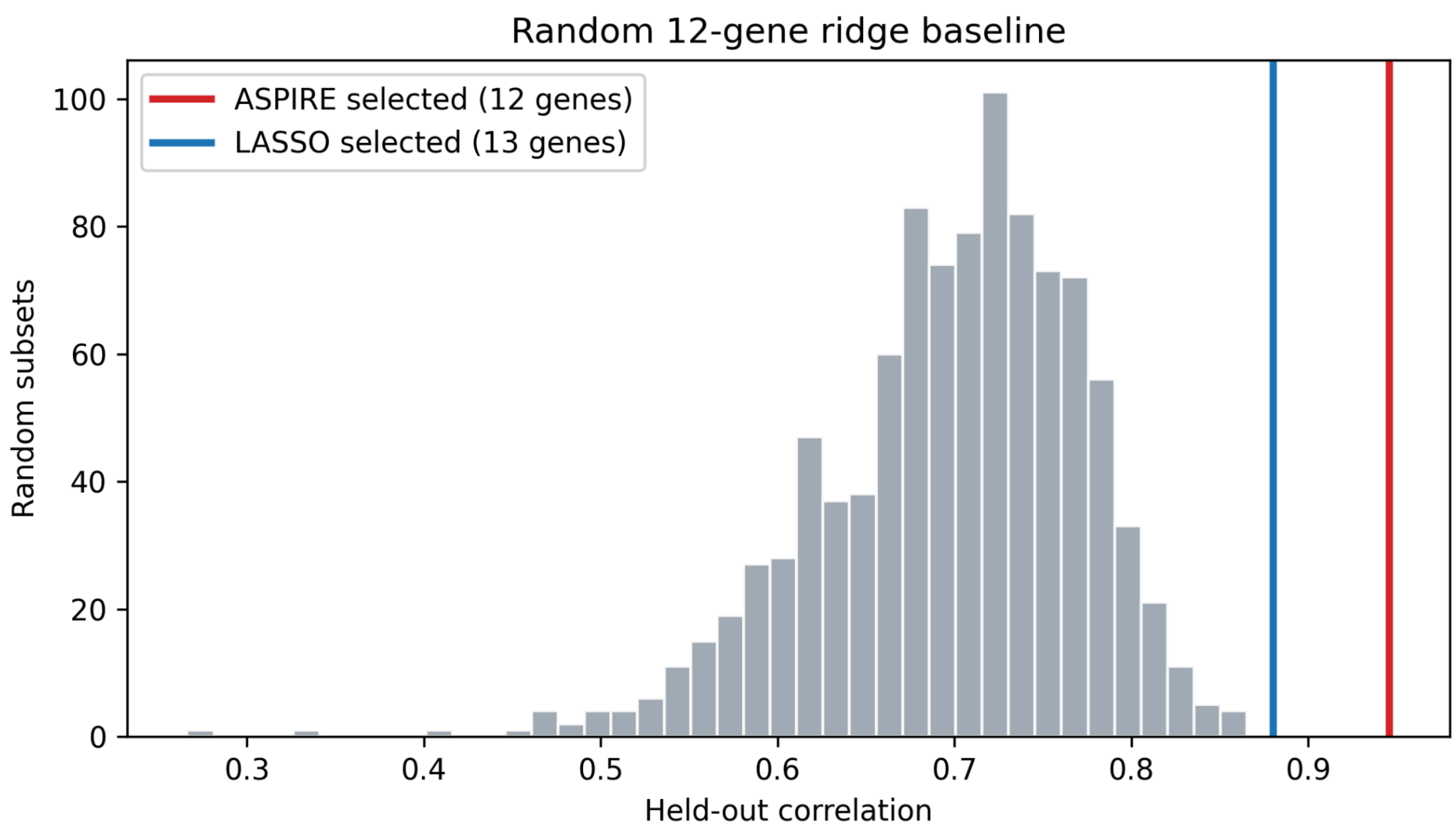


## Major comment 2

> **Reviewer #2:** *In human bulk RNA and single cells mouse model, the authors showed different genes (Fig2 and FigS1) to evaluate the prediction effects of ASPIRE compared to other tools. Could the authors consistently show relevant gene sets in different data type (for example, same gene sets for human and mouse, bulk RNA and single-cell data). Beside CLSTN1, please show a group of genes at least include PTBP1, PTBP2, ADD3, MAPT, APP, CAMK2D etc.*

**Response:** We thank the reviewer for this suggestion. We evaluated ASPIRE on a consistent panel of nine genes: CLSTN1, the six reviewer-requested genes (PTBP1, PTBP2, ADD3, MAPT, APP, CAMK2D), and two additional splicing-relevant genes (BIN1, NUMB) - across all datasets and sequencing depths. For each gene, we identified cassette exon events with

coordinates in human (hg38) and mouse (mm10) using VastDB, and computed per-exon Pearson correlations between ASPIRE-predicted PSI and the corresponding reference - either ground-truth PSI for the GTEx datasets, or MARVEL and BRIE2 PSI for the hiPSC and mouse multiple-sclerosis single-cell datasets, respectively.

The gene-level summary is now presented as a main-text figure (Fig 6; shown below, top heatmap), displaying the Pearson correlation of the top-correlated cassette exon per gene across all datasets and method combinations. Rows are genes (labelled by gene symbol; the exon shown for each gene, with coordinates, is listed in the figure legend and in Supplementary Table S2), and columns are grouped by dataset and sequencing depth. Grey cells in the BRIE2 and MARVEL columns denote exon–dataset combinations for which the respective tool did not report a PSI value, so no correlation could be computed. At full GTEx depth, ASPIRE achieved correlations above r = 0.7 for seven of nine genes, with APP (r = 0.98), ADD3 (r = 0.96), and CLSTN1 (r = 0.92) among the strongest and CAMK2D, PTBP2, and BIN1 all near r = 0.90; MAPT (r = 0.58) and NUMB (r = 0.44) were weaker. The junction-based tools also performed strongly at full depth and frequently matched or exceeded ASPIRE. For instance, for MAPT and NUMB, BRIE2 reached r = 0.89 and r = 0.95 (versus ASPIRE's r = 0.58 and r = 0.44), with MARVEL comparably high. As sequencing depth decreased, however, their agreement degraded sharply: BRIE2 correlations declined steeply (e.g., PTBP2 from r = 0.94 at full depth to r = 0.12 at 100K), and MARVEL returned no PSI estimates below full depth, whereas ASPIRE retained stable, significant correlations across all four depths. This is the expected behaviour of junction-based quantification, which depends on junction-spanning reads, and it reflects ASPIRE's intended niche: rather than surpassing these tools at high coverage, ASPIRE remains accurate in the sparse, low-depth regime where junction reads become limiting. In single-cell comparisons, correlations were generally lower, with APP showing the strongest agreement (r = 0.75 versus BRIE2 in the mouse single-cell data and r = 0.32 versus MARVEL in hiPSC).

The full per-exon results are provided as a supplementary figure (Supplementary Fig 3; shown below, bottom heatmap). Within-gene variability was substantial - for example, PTBP1 ranged from r = 0.71 for one exon to near zero for others, and a second APP cassette exon reached r = 0.82 against MARVEL in hiPSC - indicating that not all cassette exon events are equally predictable from expression profiles. The list of exons and their coordinates is provided in Supplementary Table S1, and the full correlation results are in Supplementary Table S2. These results are discussed in a new Results subsection, "Consistent evaluation across a defined gene panel" (Lines 191-224 of the revised manuscript).

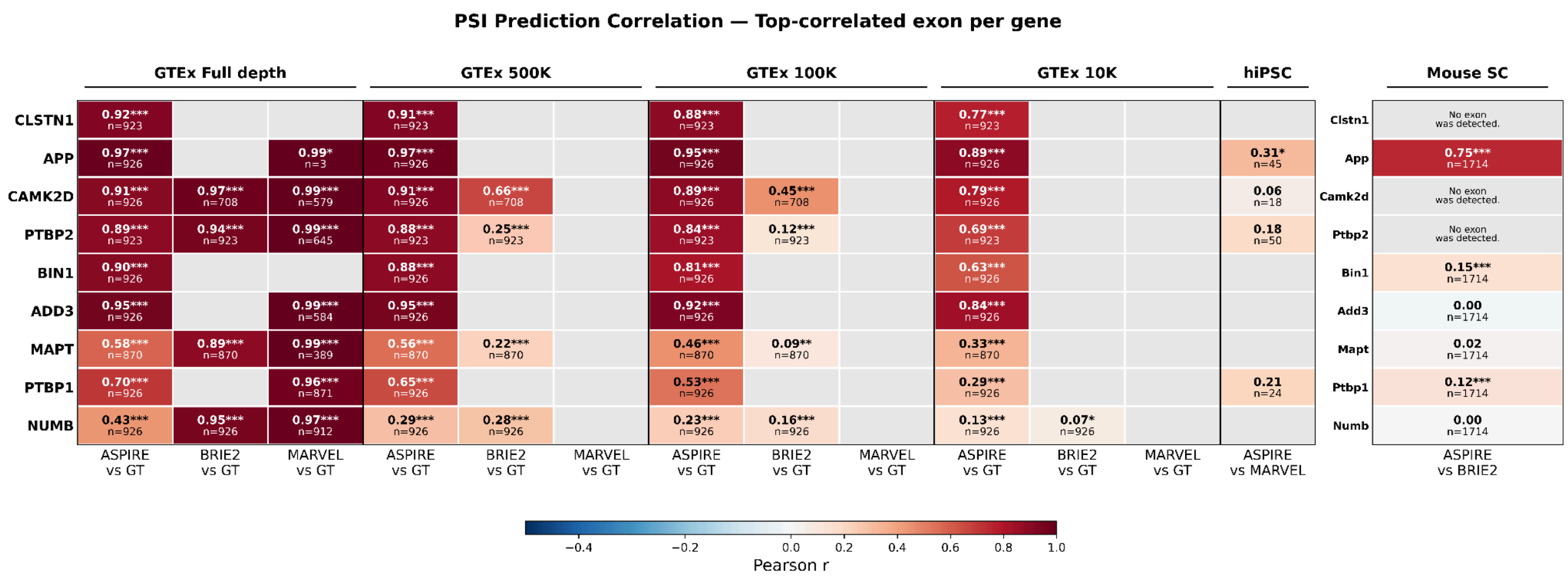
PSI Prediction Correlation — Top-correlated exon per gene
GTEx Full depth
GTEx 500K
GTEx 100K
GTEx 10K
hiPSC
Mouse SC
CLSTN1
APP
CAMK2D
PTBP2
BIN1
ADD3
MAPT
PTBP1
NUMB
Clstn1
App
Camk2d
Ptbp2
Bin1
Add3
Mapt
Ptbp1
Numb
No exon was detected.
ASPIRE vs GT
BRIE2 vs GT
MARVEL vs GT
ASPIRE vs MARVEL
ASPIRE vs BRIE2
Pearson r

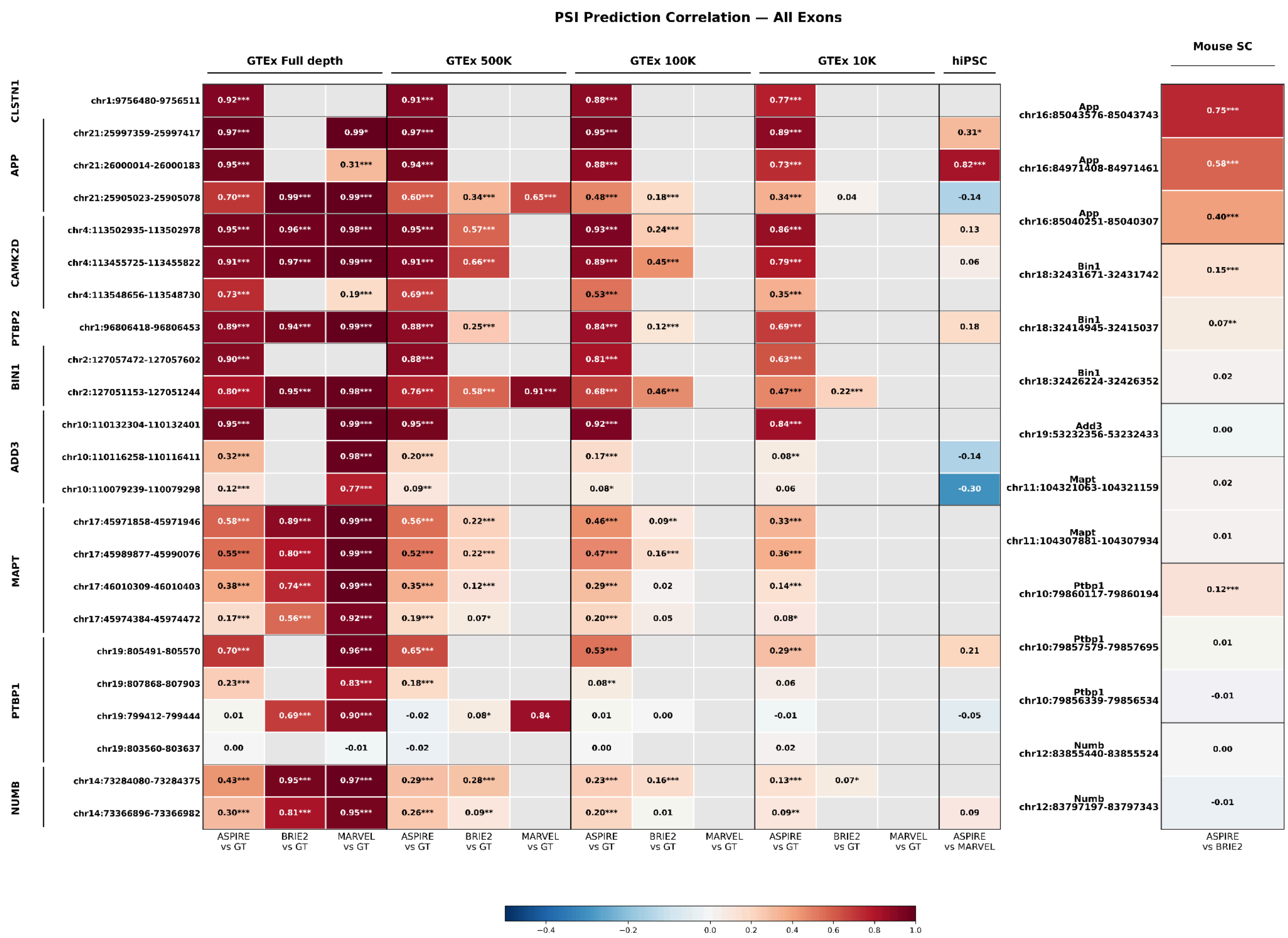
PSI Prediction Correlation — All Exons
GTEx Full depth
GTEx 500K
GTEx 100K
GTEx 10K
hiPSC
Mouse SC
CLSTN1
APP
CAMK2D
PTBP2
BIN1
ADD3
MAPT
PTBP1
NUMB
chr1:9756480-9756511
chr21:25997359-25997417
chr21:26000014-26000183
chr21:25905023-25905078
chr4:113502935-113502978
chr4:113455725-113455822
chr4:113548656-113548730
chr1:96806418-96806453
chr2:127057472-127057602
chr2:127051153-127051244
chr10:110132304-110132401
chr10:110116258-110116411
chr10:110079239-110079298
chr17:45971858-45971946
chr17:45989877-45990076
chr17:46010309-46010403
chr17:45974384-45974472
chr19:805491-805570
chr19:807868-807903
chr19:799412-799444
chr19:803560-803637
chr14:73284080-73284375
chr14:73366896-73366982
App chr16:85043576-85043743
App chr16:84971408-84971461
App chr16:85040251-85040307
Bin1 chr18:32431671-32431742
Bin1 chr18:32414945-32415037
Bin1 chr18:32426224-32426352
Add3 chr19:53232356-53232433
Mapt chr11:104321063-104321159
Mapt chr11:104307881-104307934
Ptbp1 chr10:79860117-79860194
Ptbp1 chr10:79857579-79857695
Ptbp1 chr10:79856339-79856534
Numb chr12:83855440-83855524
Numb chr12:83797197-83797343
ASPIRE vs GT
BRIE2 vs GT
MARVEL vs GT
ASPIRE vs MARVEL
ASPIRE vs BRIE2
Pearson r

## Major comment 3

> **Reviewer #2:** *For figure7, the author claimed the ASPIRE predicted better for RBP. Beside the ~1300 RBP, housekeep gene and random protein set, please show the results for Alternative-splicing relevant RBP too. For example, the author can use the gene set in this paper (https://doi.org/10.1038/s41392-024-01734-2) as a reference AS RBP.*

**Response:** We added a fourth gene pool comprising 82 AS-relevant RBPs, curated from Tao et al. (2024), to the analysis. The revised figure (now Fig.10; shown below) presents a 4×3 grid of scatter plots: rows correspond to gene pools (RNA-binding proteins, AS-relevant RBPs, housekeeping, and random) and columns to sequencing depths (Bulk, 100K, 10K). Each panel shows ASPIRE-predicted PSI versus ground-truth PSI, with Pearson r and p-value annotated. At bulk depth, the broad RBP pool achieved the highest correlation (r = 0.907), followed by random (r = 0.894), housekeeping (r = 0.890), and AS-relevant RBPs (r = 0.857). At reduced depths, the gap between gene pools widened: the AS-relevant RBP subset maintained stronger performance than housekeeping and random sets (10K: 0.495 vs 0.395 and 0.431), supporting the importance of splicing-related gene selection under sparse conditions. The gene lists for all four pools are provided in S4 Table. The Result subsection was revised accordingly (Lines 319-322, 335-338 of the revised manuscript).

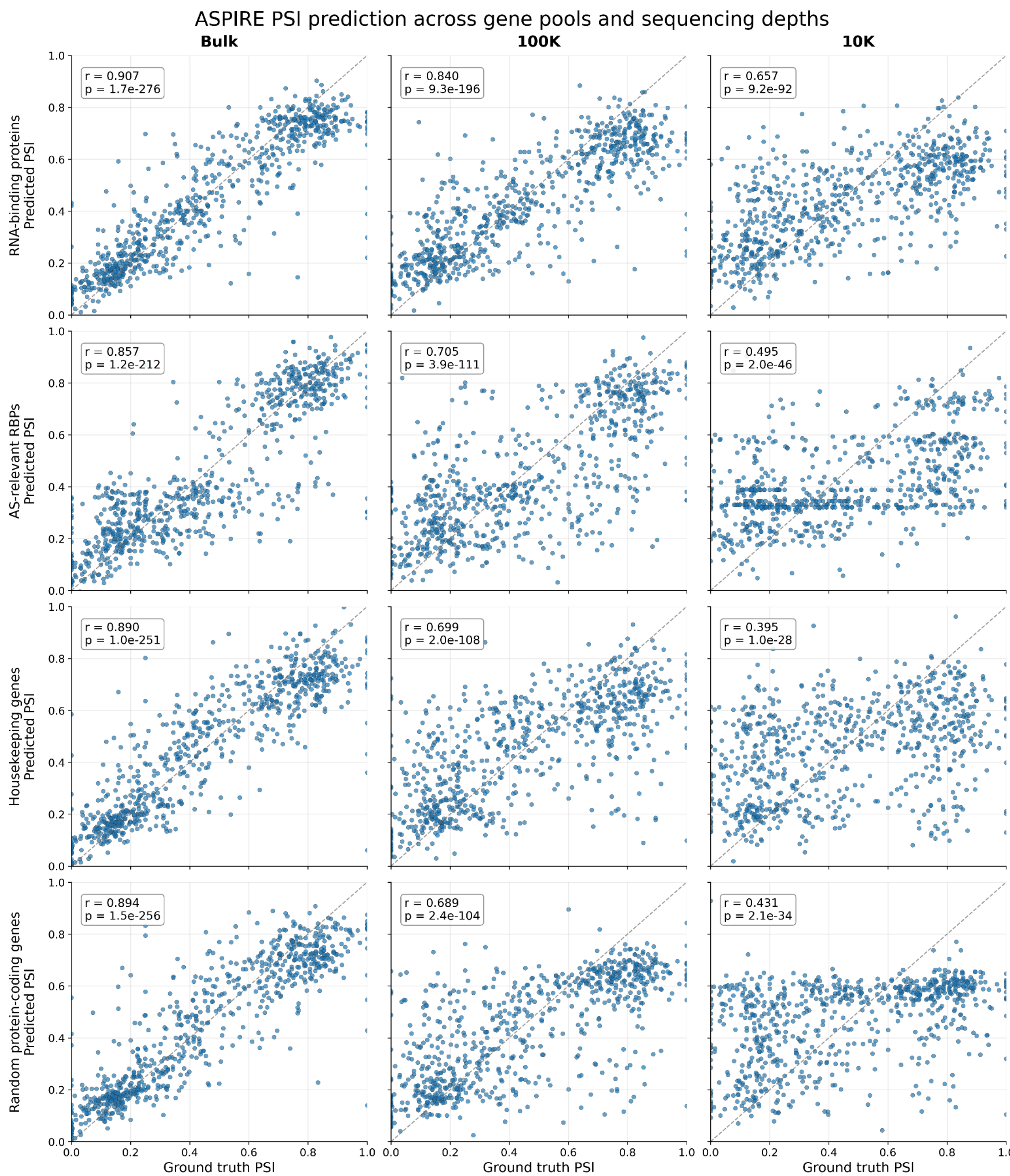


## Minor comment 1

> **Reviewer #2:** *In Line121-122, the authors introduced BRIE2 and MARVEL with citations while these two tools have been used to compare with ASPIRE at line 99 with no introductions. Please modify this in the revised manuscript.*

**Response:** We have moved the introduction and citations for BRIE2 and MARVEL to their first mention (Lines 95-96 of the revised manuscript) and removed the now-redundant re-introduction at original Line 125.

## Minor comment 2

> **Reviewer #2:** *Please display the results from line 128-143 as figure panels.*

**Response:** The MARVEL and BRIE2 results on the downsampled 100K GTEx dataset (Lines 131-144), previously reported only as text, are now presented as figure panels (Fig.5, shown below) showing the number of valid events and the distribution of per-event correlations for each tool against the bulk-derived ground truth.

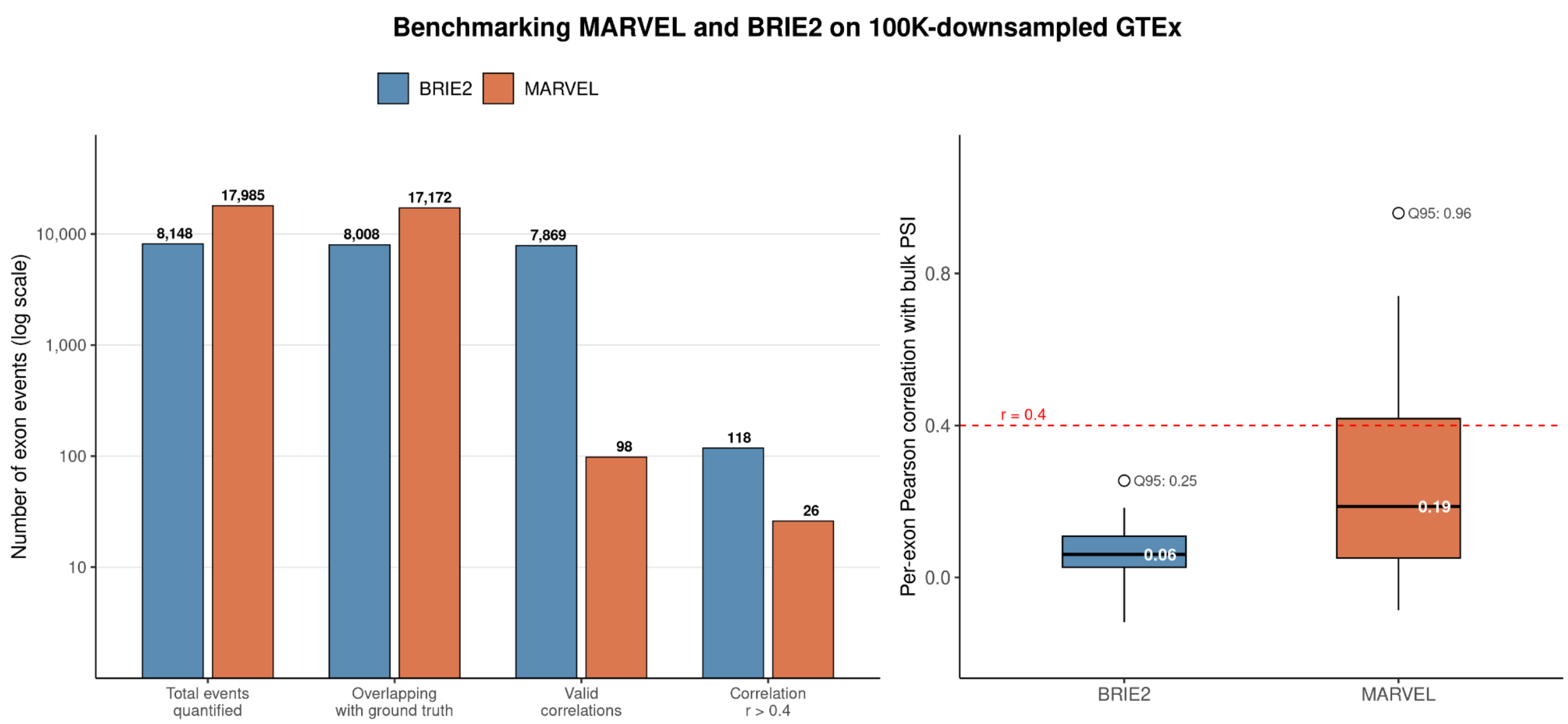


## Minor comment 3

> **Reviewer #2:** *Please annotate the corr r and p-value on each plot directly in Fig7.*

**Response:** We have revised the figure (now Fig.10, as shown in major comment 3 above) so that each scatter plot now directly reports the Pearson correlation coefficient and the corresponding two-sided p-value, computed from the plotted out-of-fold predictions.

We believe these changes substantially strengthen the revised manuscript and hope it is now suitable for publication in PLOS Computational Biology. We thank the editors and reviewers for their time and valuable input.

On behalf of all authors,

Efraim Rahamim (corresponding author), Ran Eisenberg, Eli Kopel, Miri Danan-Gotthold, Erez Y. Levanon, and Ofir Lindenbaum
