# Supplementary material for: ASPIRE: Accurate alternative splicing prediction from limited RNA sequencing data and a minimal gene set
Source: PLoS Comput Biol. 2026 Aug 28;22(8):e1014725. doi: 10.1371/journal.pcbi.1014725 (PMC13552950; doi:10.1371/journal.pcbi.1014725)
Supplement: Peer review history

# Peer review: Decision Letter 0

**DOI:** 10.1371/journal.pcbi.1014725.r001

**Authors:** Adam Ewing, Maxwell Libbrecht

31 May 2026

PCOMPBIOL-D-26-00569

ASPIRE: Accurate alternative splicing prediction from limited RNA sequencing data and a minimal gene set

PLOS Computational Biology

Dear Dr. Rahamim,

Thank you for submitting your manuscript to PLOS Computational Biology. After careful consideration, we feel that it has merit but does not fully meet PLOS Computational Biology's publication criteria as it currently stands. Therefore, we invite you to submit a revised version of the manuscript that addresses the points raised during the review process.

Please submit your revised manuscript by Jul 31 2026 11:59PM. If you will need more time than this to complete your revisions, please reply to this message or contact the journal office at ploscompbiol@plos.org. When you're ready to submit your revision, log on to https://www.editorialmanager.com/pcompbiol/ and select the 'Submissions Needing Revision' folder to locate your manuscript file.

Please include the following items when submitting your revised manuscript:

* A letter that responds to each point raised by the editor and reviewer(s). You should upload this letter as a separate file labeled 'Response to Reviewers'. This file does not need to include responses to formatting updates and technical items listed in the 'Journal Requirements' section below.

* A marked-up copy of your manuscript that highlights changes made to the original version. You should upload this as a separate file labeled 'Revised Manuscript with Track Changes'.

* An unmarked version of your revised paper without tracked changes. You should upload this as a separate file labeled 'Manuscript'.

If you would like to make changes to your financial disclosure, competing interests statement, or data availability statement, please make these updates within the submission form at the time of resubmission. Guidelines for resubmitting your figure files are available below the reviewer comments at the end of this letter.

As the corresponding author, your ORCID iD is verified in the submission system and will appear in the published article. PLOS supports the use of ORCID, and we encourage all coauthors to register for an ORCID iD and use it as well. Please encourage your coauthors to verify their ORCID iD within the submission system before final acceptance, as unverified ORCID iDs will not appear in the published article. Only the individual author can complete the verification step; PLOS staff cannot verify ORCID iDs on behalf of authors.

We look forward to receiving your revised manuscript.

Kind regards,

Adam Ewing

Academic Editor

PLOS Computational Biology

Maxwell Libbrecht

Section Editor

PLOS Computational Biology

**Additional Editor Comments:**

The reviewers' comments were overall positive. Please address the clarity of claims raised by reviewer 1 and address the questions regarding choices of gene sets raised by reviewer 2.

**Journal Requirements:**

1) We ask that a manuscript source file is provided at Revision. Please upload your manuscript file as a .doc, .docx, .rtf or .tex. If you are providing a .tex file, please upload it under the item type u2018LaTeX Source Fileu2019 and leave your .pdf version as the item type u2018Manuscriptu2019.

2) Please confirm whether your study includes live participants, human specimens or data. If so, please insert an Ethics Statement at the beginning of your Methods section, under a subheading 'Ethics Statement'. It must include:

i) The full name(s) of the Institutional Review Board(s) or Ethics Committee(s)

ii) The approval number(s), or a statement that approval was granted by the named board(s)

iii) A statement that formal consent was obtained (must state whether verbal/written) OR the reason consent was not obtained (e.g. anonymity). NOTE: If child participants, the statement must declare that formal consent was obtained from the parent/guardian.].

3) Please upload all main figures as separate Figure files in .tif or .eps format. For more information about how to convert and format your figure files please see our guidelines:

https://journals.plos.org/ploscompbiol/s/figures

4) We notice that your supplementary Figures are included in the manuscript file. Please remove them and upload them with the file type 'Supporting Information'. Please ensure that each Supporting Information file has a legend listed in the manuscript after the references list.

5) Please amend your detailed Financial Disclosure statement. This is published with the article. It must therefore be completed in full sentences and contain the exact wording you wish to be published.

1) State what role the funders took in the study. If the funders had no role in your study, please state: "The funders had no role in study design, data collection and analysis, decision to publish, or preparation of the manuscript.".

Note: If the reviewer comments include a recommendation to cite specific previously published works, please review and evaluate these publications to determine whether they are relevant and should be cited. There is no requirement to cite these works unless the editor has indicated otherwise.

**Reviewers' comments:**

Reviewer's Responses to Questions

Reviewer #1: This is a strong and useful paper with a clear goal of recovering biologically meaningful PSI estimates from limited RNA-seq data, especially under low depth and single cell like conditions. The problem is important, the motivation is solid, and the authors do a good job showing that ASPIRE performs competitively across bulk, downsampled, and external single-cell-related datasets. The addition of biologically informed feature selection and uncertainty estimation is a real strength and makes the method more interesting than a purely technical predictor.

My main suggestion is to sharpen the claims slightly. ASPIRE does not directly measure splicing, but predicts PSI from expression features that are associated with splicing behavior. That is still valuable, but the manuscript would be stronger if this distinction were stated more clearly throughout. The single cell validation is encouraging, but it still relies largely on agreement with reference computational methods rather than orthogonal ground truth, so the conclusions should stay careful in that regard.

A few simple additions could strengthen the paper. A leave-tissue-out evaluation would test whether the model generalizes beyond tissue-specific expression patterns. A stability analysis of the selected genes across folds and depths would support the biological interpretability claims. A short analysis showing that low-uncertainty predictions are indeed more reliable would also make the uncertainty framework more practical for users. It would also help to stratify performance by exon type, expression level, or PSI range, to show where the model works best.

Overall, this is a good and well executed study. It addresses a real need, is biologically important, and appears useful. I suggest a more cautious language and a few targeted analyses, then the paper would become stronger, clearer, and more accurate.

Reviewer #2: This manuscript by Eisenberg et al introduces an innovative tool called “ASPIRE”, designed to predicted alternative splicing when the sequencing reads are too sparse. They benchmarked their model in both bulk-RNA-seq data and single-cell transcriptomic data, demonstrating their tool outperformed currently widely used tool such as MARVEL and BRIE2 with just expression profiles.

However, there are some limitations regarding the evaluation dimensions and the consistence of the validation metrics. It will strengthen this study if the authors can address my comments below.

Major comments:

1. Please annotate the name and the number of the selected genes in Fig5. The author claimed a minimum of 12 genes is sufficient to build the mode. Please display the correlation between PSI for LASSO and ASPIRE. Is the 12 gene a random subset of the complete gene set?

2. In human bulk RNA and single cells mouse model, the authors showed different gene (Fig2 and FigS1) to evaluate the prediction effects of ASPIRE compared to other tools. Could the authors consistently show relevant gene sets in different data type (for example, same gene sets for human and mouse, bulk RNA and single-cell data). Beside CLSTN1., please show a gourp of genes at least include PTBP1, PTBP2, ADD3, MAPT, APP, CAMK2D etc.

3. For figure7, the author claimed the ASPIRE predicted better for RBP. Beside the ~1300 RBP, housekeep gene and random protein set, please show the results for Alternative-splicing relevant RBP too. For example, the author can use the gene set in this paper (https://doi.org/10.1038/s41392-024-01734-2) as a reference AS RBP.

Minor comments:

1. In Line121-122, the authors introduced BRIE2 and MARVEL with citations while these two tools have been used to compared with ASPIRE at line 99 with no introductions. Please modify this in the revised manuscript.

2. Please display the results from line 128-143 as figure panels.

3. Please annotate the corr r and p-value on each plot directly in Fig7.

**********

**Have the authors made all data and (if applicable) computational code underlying the findings in their manuscript fully available?**

The PLOS Data policy requires authors to make all data and code underlying the findings described in their manuscript fully available without restriction, with rare exception (please refer to the Data Availability Statement in the manuscript PDF file). The data and code should be provided as part of the manuscript or its supporting information, or deposited to a public repository. For example, in addition to summary statistics, the data points behind means, medians and variance measures should be available. If there are restrictions on publicly sharing data or code —e.g. participant privacy or use of data from a third party—those must be specified.

Reviewer #1: Yes

Reviewer #2: Yes

**********

PLOS authors have the option to publish the peer review history of their article (what does this mean?). If published, this will include your full peer review and any attached files.

If you choose “no”, your identity will remain anonymous but your review may still be made public.

**Do you want your identity to be public for this peer review?**  For information about this choice, including consent withdrawal, please see our Privacy Policy.

Reviewer #1: No

Reviewer #2: No

[NOTE: If reviewer comments were submitted as an attachment file, they will be attached to this email and accessible via the submission site. Please log into your account, locate the manuscript record, and check for the action link "View Attachments". If this link does not appear, there are no attachment files.]

**Figure resubmission:**

**Reproducibility:**

To enhance the reproducibility of your results, we recommend that authors of applicable studies deposit laboratory protocols in protocols.io, where a protocol can be assigned its own identifier (DOI) such that it can be cited independently in the future. Additionally, PLOS ONE offers an option to publish peer-reviewed clinical study protocols. Read more information on sharing protocols at https://plos.org/protocols?utm_medium=editorial-email&utm_source=authorletters&utm_campaign=protocols

# Author comment: Author response to Decision Letter 1

**DOI:** 10.1371/journal.pcbi.1014725.r002

10 Jul 2026

Attachment Submitted filename: Response to Reviewers.pdf

# Peer review: Decision Letter 1

**DOI:** 10.1371/journal.pcbi.1014725.r003

**Authors:** Adam Ewing, Maxwell Libbrecht

17 Aug 2026

Dear Mr. Rahamim,

We are pleased to inform you that your manuscript 'ASPIRE: Accurate alternative splicing prediction from limited RNA sequencing data and a minimal gene set' has been provisionally accepted for publication in PLOS Computational Biology.

Before your manuscript can be formally accepted you will need to complete some formatting changes, which you will receive in a follow up email. A member of our team will be in touch with a set of requests.

Please note that your manuscript will not be scheduled for publication until you have made the required changes, so a swift response is appreciated.

IMPORTANT: The editorial review process is now complete. PLOS will only permit corrections to spelling, formatting or significant scientific errors from this point onwards. Requests for major changes, or any which affect the scientific understanding of your work, will cause delays to the publication date of your manuscript.

Should you, your institution's press office or the journal office choose to press release your paper, you will automatically be opted out of early publication. We ask that you notify us now if you or your institution is planning to press release the article. All press must be co-ordinated with PLOS.

Thank you again for supporting Open Access publishing; we are looking forward to publishing your work in PLOS Computational Biology.

Best regards,

Adam Ewing

Academic Editor

PLOS Computational Biology

Maxwell Libbrecht

Section Editor

PLOS Computational Biology

***********************************************************

Reviewer's Responses to Questions

**Comments to the Authors:**

**Please note here if the review is uploaded as an attachment.**

Reviewer #1: Well done on the corrected version.

Reviewer #2: Eisenberg and their colleagues have fully addressed my concerns. Thanks to all authors for their efforts. I have no further comments.

**********

**Have the authors made all data and (if applicable) computational code underlying the findings in their manuscript fully available?**

The PLOS Data policy requires authors to make all data and code underlying the findings described in their manuscript fully available without restriction, with rare exception (please refer to the Data Availability Statement in the manuscript PDF file). The data and code should be provided as part of the manuscript or its supporting information, or deposited to a public repository. For example, in addition to summary statistics, the data points behind means, medians and variance measures should be available. If there are restrictions on publicly sharing data or code —e.g. participant privacy or use of data from a third party—those must be specified.

Reviewer #1: None

Reviewer #2: **No:**

**********

PLOS authors have the option to publish the peer review history of their article (what does this mean?). If published, this will include your full peer review and any attached files.

If you choose “no”, your identity will remain anonymous but your review may still be made public.

**Do you want your identity to be public for this peer review?**  For information about this choice, including consent withdrawal, please see our Privacy Policy.

Reviewer #1: No

Reviewer #2: No

# Editor report: Acceptance letter

**DOI:** 10.1371/journal.pcbi.1014725.r004

**Authors:** Adam Ewing, Maxwell Libbrecht

PCOMPBIOL-D-26-00569R1

ASPIRE: Accurate alternative splicing prediction from limited RNA sequencing data and a minimal gene set

Dear Dr Rahamim,

I am pleased to inform you that your manuscript has been formally accepted for publication in PLOS Computational Biology. Your manuscript is now with our production department and you will be notified of the publication date in due course.

The corresponding author will soon be receiving a typeset proof for review, to ensure errors have not been introduced during production. Please review the PDF proof of your manuscript carefully, as this is the last chance to correct any errors. Please note that major changes, or those which affect the scientific understanding of the work, will likely cause delays to the publication date of your manuscript.

Soon after your final files are uploaded, unless you have opted out, the early version of your manuscript will be published online. The date of the early version will be your article's publication date. The final article will be published to the same URL, and all versions of the paper will be accessible to readers.

For Research, Software, and Methods articles, you will receive an invoice from PLOS for your publication fee after your manuscript has reached the completed accept phase. If you receive an email requesting payment before acceptance or for any other service, this may be a phishing scheme. Learn how to identify phishing emails and protect your accounts at https://explore.plos.org/phishing.

Thank you again for supporting PLOS Computational Biology and open-access publishing. We are looking forward to publishing your work!

With kind regards,

Sharmila Kamatchi

PLOS Computational Biology | Carlyle House, Carlyle Road, Cambridge CB4 3DN | United Kingdom ploscompbiol@plos.org | Phone +44 (0) 1223-442824 | ploscompbiol.org | @PLOSCompBiol
